# From Dirac Structures to Port-Hamiltonian Partial Differential Equations, a Tutorial Introduction

**DOI:** 10.3390/e28030292

**Published:** 2026-03-04

**Authors:** Hans Zwart

**Affiliations:** 1 Department of Applied mathematics, University of Twente, P.O. Box 217, 7500 AE Enschede, The Netherlands; h.j.zwart@utwente.nl; 2 Faculty of Mechanical Engineering, Eindhoven University of Technology, 5600 MB Eindhoven, The Netherlands

**Keywords:** Dirac structure, port-Hamiltonian systems, partial differential equations

## Abstract

In this paper, we discuss the geometric structure, i.e., Dirac structure, underlying port-Hamiltonian systems. The paper has a tutorial character, and thus it contains questions/exercises. We start with the general definition of a Dirac structure and show that on finite-dimensional spaces, there is a simple matrix characterization. By simple examples, we show that, even in the finite-dimensional case, a Dirac structure does not guarantee the existence of solutions for an associated ordinary differential or difference equation. For associated partial differential equations, i.e., on an infinite-dimensional Dirac structure, the existence problem becomes even more challenging. We show that the spaces have to be chosen with care, but when we have shown the existence of solutions, then the Dirac structure will give us the desired properties, such as conservation of energy. The Dirac structure also implies that the associated transfer function has nice properties.

## 1. Introduction

Port-Hamiltonian systems have become a very active research area over the last decades. In books and overview articles, many results are presented, [1,2,3,4]. In the first paper on port-Hamiltonian systems [5], the underlying Dirac structure is essential for defining finite- and infinite-dimensional port-Hamiltonian systems. Although this is still the case in the first paper on a functional analytic approach to port-Hamiltonian infinite-dimensional systems, [6], in later papers following this approach, this link is hardly mentioned. For example, in [2] you will find nothing on Dirac structures. In this tutorial paper, we want to correct this.

Although our main focus will be on linear port-Hamiltonian partial differential equations, i.e., infinite-dimensional systems, we start by studying general Dirac structures in Section 2. Finite dimensional Dirac structures can be nicely characterized. We include the proofs of these characterizations, since in many papers, these are merely stated without a proof. Very good sources for the results and proofs are (chapter 2 [1]) and (section 6.6 [7]). Note that this third edition of this book contains much more and in more detail than the second edition [8]. The second half of Section 2 discusses infinite-dimensional Dirac structures. Early references on these are [5,9], a recent one is e.g., [10].

In Section 2, we also reflect upon some possible misconceptions, such as the existence of solutions for a system defined on a Dirac structure. By simple examples we show that a Dirac structure does not guarantee the existence of solutions nor stability. Furthermore, since in a Dirac structure there is no notion of time, Dirac structures can be used for continuous and discrete time systems. When we have shown the existence of solutions, then the Dirac structure will give us the desired properties, such as conservation of energy.

In several places in the manuscript, the reader may find exercises and questions. The questions will be answered shortly after they are posed, but they have the intention to let the reader stop reading, think about the topic raised, and see if she/he would have an answer to them. The exercises will be more challenging and are there to test the readers’ understanding of the material.

The material in Section 3 can be found in [2], but as said in that book you will not find the link with Dirac structures. However, the Dirac structure is the underlying structure, already hinting towards the system properties. Properties that we will discuss include the balance between internal and external power and the positive realness of the transfer function.

For the results that we cite, we tried to put the original references in. For a general introduction to port-Hamiltonian systems, including many historical results, we refer to [3, 4].

## 2. Dirac Structures

### 2.1. Motivating Example

**Figure 1:**
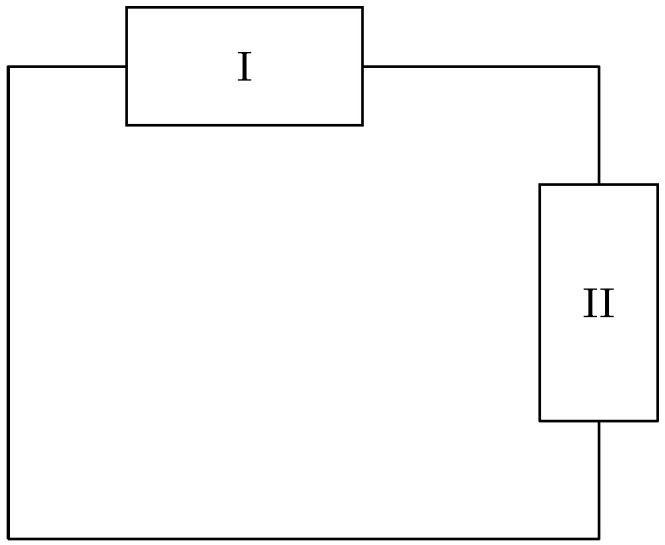
Electric network with non-specified components.

Consider the electrical network with two non-specified components, as shown in Figure 1.

Since it is an electric network, Kirchhoff’s laws apply, and so we know that the current through component I equals that of component II, and that the voltages over the components add up to zero, i.e.,

$$I_{I}=I_{II},and\;V_{I}+V_{II}=0.$$

We can write these in a matrix-vector notation

(1) $$\left(\begin{array}{cc} 1 & -1\\ 0 & 0 \end{array}\right)\left(\begin{array}{c} I_{I}\\ I_{II} \end{array}\right)+\left(\begin{array}{cc} 0 & 0\\ 1 & 1 \end{array}\right)\left(\begin{array}{c} V_{I}\\ V_{II} \end{array}\right)=\left(\begin{array}{c} 0\\ 0 \end{array}\right).$$

Since the network in Figure 1 is a closed system, no energy will leave. Thus, we have that the total change in energy, i.e., the power, will be zero. The power of an electrical network is current times voltage, and so it should hold

(2) $$I_{I}V_{I}+I_{II}V_{II}=0.$$

It is easy to see that this holds for any quadruple satisfying (1). Note that if we were to reorder the components in the vectors and adjust the associated matrices in (1) accordingly, then (2) would still hold. For instance, we could have written Kirchhoff’s laws as

(3) $$\left(\begin{array}{cc} 1 & 0\\ 0 & 1 \end{array}\right)\left(\begin{array}{c} I_{I}\\ V_{II} \end{array}\right)+\left(\begin{array}{cc} 0 & -1\\ 1 & 0 \end{array}\right)\left(\begin{array}{c} V_{I}\\ I_{II} \end{array}\right)=\left(\begin{array}{c} 0\\ 0 \end{array}\right).$$

In both cases we see that the total power equals the inner product between the two (voltage-current) vectors.

In conclusion, we see that independently of how we choose the components of an electric network, the power will always be conserved. Of course, this is the case in other fields of physics as well. No matter which model of an (undamped) pendulum we decide to use, we want conservation of energy, or equivalently, the total power to be zero. This has led to the introduction of a special structure, the Dirac structure. In the following sections, we define it in a very general way. This will allow us to use the results for systems described by ordinary and partial differential equations, which are formulated as continuous and/or discrete-time systems.

### 2.2. General Definitions and Properties

Motivated by the conservation of energy property of (undamped) systems, we introduce Dirac structures in their most general form.

Let E and F be two real (complex) two linear spaces with the bilinear product

〈f∣e〉∈R(or C).

We assume that this product is *non-degenerate*, that is

$$\begin{array}{cc} \langle f|e\rangle = & 0\;\forall e\in E\Rightarrow f=0,\;and\\ \langle f|e\rangle = & 0\;\forall f\in F\Rightarrow e=0. \end{array}$$

E is called the *effort space* and F is the *flow space*. The *bond space* B is defined as F×E. On the bond space B=F×E we define the symmetrized pairing

(4) $${\langle \left(\begin{array}{c} f_{1}\\ e_{1} \end{array}\right),\left(\begin{array}{c} f_{2}\\ e_{2} \end{array}\right)\rangle }_{B}=\langle f_{2}|e_{1}\rangle +\langle f_{1}|e_{2}\rangle .$$

For V⊆B we define

(5) $$V^{⊥}=\left\{\left(\begin{array}{c} f_{1}\\ e_{1} \end{array}\right)\in B|{\langle \left(\begin{array}{c} f_{1}\\ e_{1} \end{array}\right),\left(\begin{array}{c} f_{2}\\ e_{2} \end{array}\right)\rangle }_{B}=0\;for\;all\;(\begin{array}{c} f_{2}\\ e_{2} \end{array})\in V\right\}.$$

**Definition** **1.** *The linear subspace D of B is a Dirac structure if $D^{⊥}=D$.*

If D is a Dirac structure, then we have that any element $(\begin{array}{c} f\\ e \end{array})\in D$ is also an element of $D^{⊥}$. Hence, by (4) and (5) there holds

(6) $$0={\langle \left(\begin{array}{c} f\\ e \end{array}\right),\left(\begin{array}{c} f\\ e \end{array}\right)\rangle }_{B}=2\langle f|e\rangle =0\;for\;all\left(\begin{array}{c} f\\ e \end{array}\right)\in D.$$

So we find that 〈f|e〉=0 for all $(\begin{array}{c} f\\ e \end{array})\in D$. This has (may have) the interpretation of power conservation, as we will see later.

### 2.3. Dirac Structures, Finite-Dimensional

For finite-dimensional spaces, the following gives a very useful characterisation of a Dirac structure, see (Section 1.2 [11]) or [12].

**Lemma** **1.** *For $F=E=R^{n}$ with*

$$\langle f|e\rangle =f^{⊤}e$$

*we have that D is a Dirac structure if and only if there exist two n×n matrices F and E, such that*

*1.*  *$D=\mathrm{ran}(\begin{array}{c} F\\ E \end{array})$;*
*2.*  *The matrix $\left(\begin{array}{c} F\\ E \end{array}\right)$ has full rank (rank equals n);*
*3.*  *$F^{⊤}E+E^{⊤}F=0$, or in other words $F^{⊤}E$ is skew-adjoint (anti-symmetric).*

**Proof.**  Assume that $D\subset R^{n}\times R^{n}$ is a Dirac structure. Then, since it is a linear subspace, there exist matrices *F* and *E* of size (n×m) such that $D=\mathrm{ran}\left(\begin{array}{c} F\\ E \end{array}\right)$ and $\left(\begin{array}{c} F\\ E \end{array}\right)$ is of full rank (rank equals *m*).

Using this, we have that $\left(\begin{array}{c} f_{2}\\ e_{2} \end{array}\right)\in D^{⊥}$ if and only if $\left(\begin{array}{c} f_{2}\\ e_{2} \end{array}\right)⊥\mathrm{ran}\left(\begin{array}{c} F\\ E \end{array}\right)$. This relation is a linear equation with 2n unknowns and *m* conditions. Hence, the dimension of the solution set is 2n−m-dimensional. However, since $D^{⊥}$ equals D we have 2n−m=m. Thus, m=n. So we have shown item 1. and 2. of the lemma.

To show the third item, we note that 〈f|e〉=0 for all $\left(\begin{array}{c} f\\ e \end{array}\right)\in D$, see (6). Since $D=\mathrm{ran}\left(\begin{array}{c} F\\ E \end{array}\right)$ this is equivalent to $\ell^{⊤}F^{⊤}E\ell =0$ for all $\ell \in R^{n}$. Thus, $\ell^{⊤}\left[F^{⊤}E+E^{⊤}F\right]\ell =0$. Since $F^{⊤}E+E^{⊤}F$ is symmetric, we conclude that $F^{⊤}E+E^{⊤}F=0$.

Next we prove the other implication. Let $D=\mathrm{ran}\left(\begin{array}{c} F\\ E \end{array}\right)$ with $\left(\begin{array}{c} F\\ E \end{array}\right)$ a 2n×n matrix of rank *n* satisfying $F^{⊤}E+E^{⊤}F=0$. We have to show that D is a Dirac structure.

Given $\left(\begin{array}{c} f_{2}\\ e_{2} \end{array}\right)\in D$ we have for any $\left(\begin{array}{c} f_{1}\\ e_{1} \end{array}\right)\in D$ that

$$\begin{array}{cc} {\langle \left(\begin{array}{c} f_{1}\\ e_{1} \end{array}\right),\left(\begin{array}{c} f_{2}\\ e_{2} \end{array}\right)\rangle }_{B}= & \;\langle f_{2}|e_{1}\rangle +\langle f_{1}|e_{2}\rangle \\ = & \;\ell_{2}^{⊤}F^{⊤}E\ell_{1}+\ell_{1}^{⊤}F^{⊤}E\ell_{2}\\ = & \;\ell_{2}^{⊤}F^{⊤}E\ell_{1}+\ell_{2}^{⊤}E^{⊤}F\ell_{1}=0. \end{array}$$

By definition, see (5), this implies that $\left(\begin{array}{c} f_{2}\\ e_{2} \end{array}\right)\in D^{⊥}$. Since $\left(\begin{array}{c} f_{2}\\ e_{2} \end{array}\right)$ was an arbitrary element in D and we have shown that $D\subseteq D^{⊥}$.

By construction $\dim(D^{⊥})=2n-n$, namely, the dimension of the space minus the number of conditions. So $\dim\left(D^{⊥}\right)=n=\dim\left(D\right)$. Combining this with the inclusion $D\subseteq D^{⊥}$, we find that $D=D^{⊥}$. □

**Exercise** **1.** *In this exercise we consider for $Q\in R^{n\times n}$ the bilinear product on $R^{n}\times R^{n}$*

(7) $$\langle f|e\rangle :=f^{⊤}Qe.$$

*1.*  *Under which condition on Q is the bilinear product (7) non-degenerate?*
*2.*  *Let Q be such that the bilinear product (7) is non-degenerate. Let $D\subset R^{n}\times R^{n}$ be written as $D=\mathrm{ran}\left(\begin{array}{c} F\\ E \end{array}\right)$. Formulate (equivalent) conditions on F and E such that D is a Dirac structure with respect to the bilinear product (7).*

From now on, we assume the finite-dimensional case, i.e., $F=E=R^{n}$ with $\langle f|e\rangle =f^{⊤}e$. For these effort and flow spaces and that bilinear product, we have seen that every Dirac structure can be written as $D=\mathrm{ran}\left(\begin{array}{c} F\\ E \end{array}\right)$ with $\left(\begin{array}{c} F\\ E \end{array}\right)$ a 2n×n matrix of rank *n* satisfying $F^{⊤}E+E^{⊤}F=0$. This is known as the *image representation*.

**Lemma** **2.** *Let a Dirac structure on $R^{n}\times R^{n}$ be given as $D=\mathrm{ran}\left(\begin{array}{c} F\\ E \end{array}\right)$ with the above conditions on E,F. Then, D has the kernel representation*

$$D=\ker\left(\begin{array}{cc} E^{⊤} & F^{⊤} \end{array}\right).$$

**Proof.**  For $\left(\begin{array}{c} f\\ e \end{array}\right)\in D$ we have

$$\left(\begin{array}{cc} E^{⊤} & F^{⊤} \end{array}\right)\left(\begin{array}{c} f\\ e \end{array}\right)=\left(\begin{array}{cc} E^{⊤} & F^{⊤} \end{array}\right)\left(\begin{array}{c} F\\ E \end{array}\right)\ell =0.$$

Hence, $D\subseteq \ker\left(\begin{array}{cc} E^{⊤} & F^{⊤} \end{array}\right)$. By checking dimensions, we find that these sets are equal. □

**Exercise** **2.** *Under which condition(s) is $D=\ker\left(\begin{array}{cc} E_{1} & F_{1} \end{array}\right)$ a Dirac structure? Furthermore, what is its image representation?*

Next to the characterisation given in Lemma 1, we have the following alternative characterisation of a Dirac structure, see (Proposition 1.3.2 [11]).

**Lemma** **3.** *$D=\mathrm{ran}\left(\begin{array}{c} F\\ E \end{array}\right)$ is a Dirac structure if and only if there exists a unitary matrix $\Theta \in R^{n\times n}$ such that $D=\mathrm{ran}\left(\begin{array}{c} -I+\Theta \\ I+\Theta \end{array}\right)$.*

**Proof.**  Using, from Lemma 1, that $F^{T}E+E^{T}F=0$, we find that

(8) $${\left(E+F\right)}^{T}\left(E+F\right)={\left(E-F\right)}^{T}\left(E-F\right).$$

So, if (E−F)v=0, then (E+F)v=0, and thus Ev=Fv=0. Since $\left(\begin{array}{c} F\\ E \end{array}\right)$ has full rank, we find v=0 and so we have that E−F is invertible. Define $\Theta =\left(E+F\right){\left(E-F\right)}^{-1}$, then (8) implies that Θ is unitary. Now $D=\mathrm{ran}\left(\begin{array}{c} F\\ E \end{array}\right)=\mathrm{ran}\left(\begin{array}{c} 2F{\left(E-F\right)}^{-1}\\ 2E{\left(E-F\right)}^{-1} \end{array}\right)=\mathrm{ran}\left(\begin{array}{c} -I+\Theta \\ I+\Theta \end{array}\right)$.

Next, assume that $\Theta \in R^{n\times n}$ is unitary. To show that $D=\mathrm{ran}\left(\begin{array}{c} -I+\Theta \\ I+\Theta \end{array}\right)$ is a Dirac structure, we check the conditions in Lemma 1.

If $\left(\begin{array}{c} -I+\Theta \\ I+\Theta \end{array}\right)$ would not have full rank, then, −I+Θ and I+Θ would have a mutual non-zero element in their kernels. It is straightforward to see that this is not possible. Hence, it remains to check condition 3. of Lemma 1. We see that

$$\begin{array}{cc} {\left(-I+\Theta \right)}^{⊤} & \left(I+\Theta \right)+{\left(I+\Theta \right)}^{⊤}\left(-I+\Theta \right)\\ = & -I+\Theta^{⊤}-\Theta +\Theta^{⊤}\Theta -I+\Theta -\Theta^{⊤}+\Theta^{⊤}\Theta =0, \end{array}$$

where we have used that Θ is unitary. So $D:=\mathrm{ran}\left(\begin{array}{c} -I+\Theta \\ I+\Theta \end{array}\right)$ satisfies the necessary conditions in Lemma 1 and thus is a Dirac structure. □

Combining the previous two lemmata gives another characterisation of a Dirac structure.

**Exercise** **3.** *Prove that $D=\mathrm{ran}\left(\begin{array}{c} F\\ E \end{array}\right)$ is a Dirac structure if and only if there exists a unitary matrix $\Theta \in R^{n\times n}$ such that $D=\ker\left(I+\Theta ,\;I-\Theta \right)$.*

### 2.4. Dirac Structures, Dynamical Interpretation

In this section, we study dynamical systems defined on a Dirac structure. As before, we assume that $F=E=R^{n}$ with the bilinear product $\langle f|e\rangle =f^{⊤}e$.

**Example** **1.** *We take F=J, E=I, with $J^{⊤}=-J$. Then, by lemmatas 1 and 2*

$$D=\ker\left(\begin{array}{cc} I^{⊤} & J^{⊤} \end{array}\right)=\ker\left(\begin{array}{cc} I & -J \end{array}\right)$$

*defines a Dirac structure.*

*So, the solutions of $\dot{x}\left(t\right)=JHx\left(t\right)$ ($H=H^{⊤}\in R^{n\times n}$) can be seen as $\left(\begin{array}{c} f\\ e \end{array}\right)=\left(\begin{array}{c} \dot{x}\left(t\right)\\ Hx(t) \end{array}\right)\in D$ and satisfy*

(9) $$\frac{d}{dt}\left[\frac{1}{2}x{\left(t\right)}^{⊤}Hx\left(t\right)\right]=\dot{x}{\left(t\right)}^{⊤}Hx\left(t\right)=f^{⊤}e=0.$$

*Thus, $H\left(t\right):=\frac{1}{2}x{\left(t\right)}^{⊤}Hx\left(t\right)$ is constant along solutions of the differential equation.*

Note that in the above example, we choose *f* as $\dot{x}\left(t\right)$. In the literature, you may also find the choice $f=-\dot{x}\left(t\right)$, giving equivalent results. In the example, we only needed that H is symmetric. From the fact that *H* is constant along solutions, one would be tempted to conclude that the system is stable, but this need not be the case.

**Exercise** **4.** *In the previous example, take n=2 and $J=\left(\begin{array}{cc} 0 & 1\\ -1 & 0 \end{array}\right)$. Show that, for H=diag(1,−1) the differential equation $\dot{x}\left(t\right)=JHx\left(t\right)$ is unstable, although $\frac{1}{2}x{\left(t\right)}^{⊤}Hx\left(t\right)$ is constant along solutions.*

The previous example can be extended to non-linear o.d.e.’s.

**Example** **2.** *Let a Dirac structure D be given as $D=\mathrm{ran}\left(\begin{array}{c} F\\ E \end{array}\right)$ with $\left(\begin{array}{c} F\\ E \end{array}\right)$ a 2n×n matrix of rank n satisfying $F^{⊤}E=-E^{⊤}F$.*

*With the $C^{1}$-function $H:R^{n}\mapsto R$, we define the implicit differential equation*

(10) $$\left(\begin{array}{c} \dot{x}\left(t\right)\\ \frac{\partial H}{\partial x}\left(x\left(t\right)\right) \end{array}\right)\in D.$$

*Then, similar as in (9), we have that $\frac{d}{dt}H\left(x\left(t\right)\right)=0$ along solutions.*

*Using the kernel representation of D, we have that the implicit differential Equation (10) can be made explicitly as*

(11) $$E^{⊤}\dot{x}\left(t\right)=-F^{⊤}\frac{\partial H}{\partial x}\left(x\left(t\right)\right).$$

Note that since *E* does not need to be invertible, the differential Equation (11) could be a *Differential Algebraic Equation, (DAE)*. Hence, this differential equation does not need to have solutions (for all initial conditions). For instance, take E=0 and F=I, then the (11) becomes $0=-\frac{\partial H}{\partial x}\left(x\left(t\right)\right)$.

**Exercise** **5.** *In the introduction, we have treated an electric network with non-specified components. In this exercise, we specify these components, and study the existence of solutions of the associated differential equations. However, before doing so, we relate (1) and (3) to a Dirac structure.*

**Figure 2:**
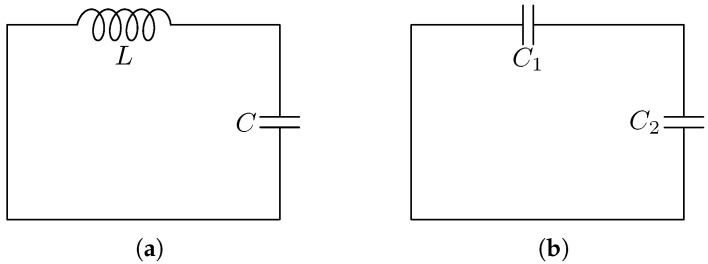
Electric network of Figure 1 with specified components. (**a**) As an LC network. (**b**) As a CC network.

*1.*  *Show that (1) defines a Dirac structure on $R^{2}\times R^{2}$ with f as the first vector, i.e, $f=\left(\begin{array}{c} I_{I}\\ I_{II} \end{array}\right)$, and e the second.*
*2.*  *Show that (3) defines a Dirac structure on $R^{2}\times R^{2}$ with f as the first vector, i.e, $f=\left(\begin{array}{c} I_{I}\\ V_{II} \end{array}\right)$, and e the second.*
*3.*  *By using the modelling equations $C\frac{dV_{C}}{dt}=I_{C}$ and $L\frac{dI_{L}}{dt}=V_{L}$ for the capacitor and the inductor, respectively, derive a model, like (11), for the network in Figure 2a. Show that your differential equation has for every initial condition in $R^{2}$ a unique solution.*
*4.*  *By using the modelling equation $C\frac{dV_{C}}{dt}=I_{C}$ for the capacitors, derive a model, like (11) for the network in Figure 2b. Show that your differential equation does not have a solution for every initial condition in $R^{2}$.*

So, the examples show that a differential equation defined on a Dirac structure does not guarantee existence nor stability. In the definition of a Dirac structure there was no notion of time, this came in via conditions like (10), stating that we want a trajectory t∈T=[0,∞)↦x(t) such that (10) is satisfied. Here we already made a choice, alternative choices would be T=R or $T=[0,t_{1})$, etc. Moreover, we do not have to choose a continuous time axis to define a system on a Dirac structure.

**Example** **3.** *We take the same Dirac structure as in Example 1. Thus,*

(12) $$D=\ker\left(\begin{array}{cc} I & -J \end{array}\right)$$

*with $J\in R^{n\times n}$ satisfying $J^{⊤}=-J$. Furthermore, we take $H\in R^{n\times n}$ satisfying $H=H^{⊤}$.*

*The solutions of the implicit difference equation*

(13) $$x\left(n+1\right)-x\left(n\right)=JH\left[x(n+1)+x(n)\right],\;n\in N$$

*can be seen as $\left(\begin{array}{c} f\\ e \end{array}\right)=\left(\begin{array}{c} x(n+1)-x(n)\\ H\left[x(n+1)+x(n)\right] \end{array}\right)\in D$ and satisfy*

$$\begin{array}{cc} x{\left(n+1\right)}^{T}H & x(n+1)-x(n)Hx(n)=\\ \; & {\left[x(n+1)-x(n)\right]}^{T}H\left[x(n+1)+x(n))\right]=f^{⊤}e=0. \end{array}$$

*Thus, $H\left(n\right):=x{\left(n\right)}^{⊤}Hx\left(n\right)$ is constant along solutions of the difference in Equation (13).*

Note that if I−JH is invertible, then the implicit difference in Equation (13) can be made explicit. Namely, to

$$x\left(n+1\right)={\left(I-JH\right)}^{-1}\left(I+JH\right)x\left(n\right).$$

**Exercise** **6.** *Prove that if $J,H\in R^{n\times n}$ satisfy $J^{⊤}=-J$, $H=H^{⊤}$ and H≥0, then the matrix I−JH is invertible.*

In the previous examples of dynamical systems, we choose *f* to be the change in the state variable *x*. However, this is not dictated by the Dirac structure. Other choices are possible.

**Example** **4.** *We take the same Dirac structure as in Example 3, but now we split our effort and flow space, and choose J in a special form*

$$f=\left(\begin{array}{c} \phi_{1}\\ \phi_{2} \end{array}\right),e=\left(\begin{array}{c} \varepsilon_{1}\\ \varepsilon_{2} \end{array}\right),J=\left(\begin{array}{cc} J_{11} & J_{12}\\ -J_{12}^{⊤} & 0 \end{array}\right)$$

*with $J_{11}$ skew-symmetric. For $\phi_{1}=\dot{x}\left(t\right)$, $\varepsilon_{2}=R\phi_{2}$, and $\varepsilon_{1}=Hx\left(t\right)$ with H and R symmetric matrices, the relation f=Je becomes*

$$\left(\begin{array}{c} \dot{x}\left(t\right)\\ \phi_{2} \end{array}\right)=\left(\begin{array}{cc} J_{11} & J_{12}\\ -J_{12}^{⊤} & 0 \end{array}\right)\left(\begin{array}{c} Hx(t)\\ R\phi_{2} \end{array}\right).$$

*Hence, x satisfies $\dot{x}\left(t\right)=\left(J_{11}-J_{12}RJ_{12}^{⊤}\right)Hx\left(t\right)$. Furthermore,*

(14) $$\dot{x}{\left(t\right)}^{⊤}Hx\left(t\right)+\phi_{2}^{⊤}R\phi_{2}=f^{⊤}e=0.$$

*When R≥0, this implies that $\dot{x}{\left(t\right)}^{⊤}Hx\left(t\right)\leq 0$. This we can see as dissipation of $H\left(t\right)=\frac{1}{2}x{\left(t\right)}^{⊤}Hx\left(t\right)$, i.e., $\dot{H}\left(t\right)\leq 0$.*

*Thus, although the Dirac structure gives conservation, i.e., $f^{⊤}e=0$, it does allow us to build differential equations with dissipation.*

In the previous example, we showed how systems with dissipation can be linked to a Dirac structure, the following example shows that the same is possible for a system with inputs and outputs.

**Example** **5.** *We take the same Dirac structure as in Example 3, but now we split our effort and flow space, and choose J as*

$$f=\left(\begin{array}{c} \phi_{1}\\ \phi_{2} \end{array}\right),e=\left(\begin{array}{c} \varepsilon_{1}\\ \varepsilon_{2} \end{array}\right),J=\left(\begin{array}{cc} J_{11} & B\\ -B^{⊤} & -J_{22} \end{array}\right)$$

*with $J_{11}$ and $J_{22}$ skew-symmetric. For $\phi_{1}=\dot{x}\left(t\right)$, $\phi_{2}=-y\left(t\right)$, $\varepsilon_{2}=u\left(t\right)$ and $\varepsilon_{1}=Hx\left(t\right)$, f=Je becomes*

$$\left(\begin{array}{c} \dot{x}\left(t\right)\\ -y(t) \end{array}\right)=\left(\begin{array}{cc} J_{11} & B\\ -B^{⊤} & -J_{22} \end{array}\right)\left(\begin{array}{c} Hx(t)\\ u(t) \end{array}\right).$$

*So, the input-state-output system*

(15) $$\dot{x}\left(t\right)=J_{11}Hx\left(t\right)+Bu\left(t\right)$$

(16) $$y(t)=B^{⊤}Hx\left(t\right)+J_{22}u\left(t\right).$$

*Similar to before, we see that $f^{⊤}e=0$ implies that along solutions of (15) and (16) there holds*

(17) $$\dot{x}{\left(t\right)}^{⊤}Hx\left(t\right)-y{\left(t\right)}^{⊤}u\left(t\right)=0.$$

**Figure 3:**
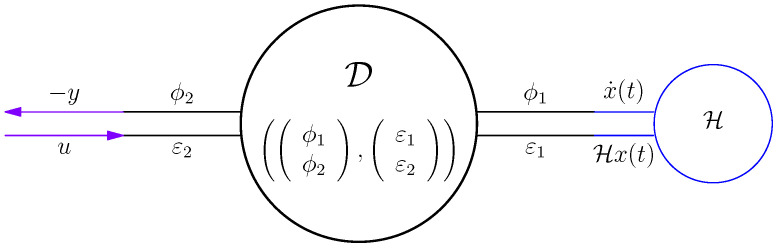
Dirac structure connected to storage H, input *u*, and output *y*.

*This has the interpretation that the energy lost/gained by internal variables, x equals the energy supplied/taken by the external variables u,y. Graphically, (15) and (16) are represented by Figure 3.*

*The systems (15) and (16) are (standard) examples of a port-Hamiltonian system, with $H\left(t\right)=\frac{1}{2}x{\left(t\right)}^{⊤}Hx\left(t\right)$ the Hamiltonian and (u,y) the external ports.*

### 2.5. Dirac Structures, Infinite-Dimensional

So, far we have considered E and F to be finite-dimensional, i.e., $R^{n}$. Other (finite-dimensional) choices are possible, e.g., a tangent space and co-tangent space, see e.g., subsection 2.4.4 of [1]. Since the dimension is not “present” in the definition of a Dirac structure, we can take E and F to be *infinite-dimensional*, as was already done in [5].

There are many infinite-dimensional spaces, i.e., function and/or sequence spaces, and so we take a simpler approach, and try to see if we can come up with an example in which

$$V=\left\{\left(\begin{array}{c} f\\ e \end{array}\right)|f=Je\right\}$$

is an infinite-dimensional Dirac structure.

**Question** **1.** *If f and e are two (scalar) functions on* Ω*, what would be a logical choice for 〈f∣e〉?*

A choice is

(18) $$\langle f|e\rangle =\int_{\Omega }f\left(\zeta \right)e\left(\zeta \right)d\zeta .$$

For simplicity, we take Ω=[a,b]⊆R.

**Question** **2.** *Taking the bilinear product (18), can you think of an J such that {f=Je} is a Dirac structure?*

*Note that you need to satisfy*

(19) $$\langle f|e\rangle =\int_{a}^{b}f\left(\zeta \right)e\left(\zeta \right)d\zeta =\int_{a}^{b}\left(Je\right)\left(\zeta \right)e\left(\zeta \right)d\zeta =0.$$

A candidate could be $Je=\frac{de}{d\zeta }$. To check (19), we calculate the bilinear product of *e* and Je

$$\begin{array}{cc} \langle f|e\rangle = & \;\int_{a}^{b}\left(Je\right)\left(\zeta \right)e\left(\zeta \right)d\zeta =\int_{a}^{b}\frac{de}{d\zeta }\left(\zeta \right)e\left(\zeta \right)d\zeta \\ = & \;\int_{a}^{b}\frac{1}{2}\frac{d}{d\zeta }\left(e{\left(\zeta \right)}^{2}\right)d\zeta =\frac{1}{2}e{\left(b\right)}^{2}-\frac{1}{2}e{\left(a\right)}^{2}. \end{array}$$

So, this is only zero when we put (extra) conditions on *e*. For instance, e(b)=e(a)=0, e(b)=e(a), or e(b)=−e(a). For the first two choices, you are asked to check whether it is a Dirac structure.

**Question** **3.** *Given F=C(a,b) and $E=C^{1}\left(a,b\right)$. Is*

$$D_{00}=\left\{\left(\begin{array}{c} f\\ e \end{array}\right)\in F\times E|f=\frac{de}{d\zeta },e\left(a\right)=0=e\left(b\right)\right\}$$

*a Dirac structure?*

**Answer**: We start by calculating $D_{00}^{⊥}$. We have that $\left(\begin{array}{c} f_{2}\\ e_{2} \end{array}\right)\in D_{00}^{⊥}$ if and only if

$$\langle \left(\begin{array}{c} f_{2}\\ e_{2} \end{array}\right)|\left(\begin{array}{c} f\\ e \end{array}\right)\rangle =0\;\forall \left(\begin{array}{c} f\\ e \end{array}\right)\in D_{00}.$$

By the definition of the bilinear product (19) and $D_{00}$, this is equivalent to

$$\int_{a}^{b}f_{2}\left(\zeta \right)e\left(\zeta \right)+e_{2}\left(\zeta \right)\frac{de}{d\zeta }\left(\zeta \right)d\zeta =0\;\forall e\in C^{1}\left(a,b\right).$$

Since $e_{2}$ is a $C^{1}$-function, we can apply the integration by parts formula. This gives

(20) $$\int_{a}^{b}\left[f_{2}\left(\zeta \right)-\frac{de_{2}}{d\zeta }\left(\zeta \right)\right]e\left(\zeta \right)d\zeta =0\;\forall e\in C^{1}\left(a,b\right),$$

where we have used that e(a)=e(b)=0. Since $f_{2}$ and $\frac{de_{2}}{d\zeta }$ are continuous, the above condition implies that $f_{2}\left(\zeta \right)=\frac{de_{2}}{d\zeta }\left(\zeta \right)$. So, we only satisfy the defining conditions of $D_{00}$ partially. In particular, the pair $f_{2}=0,e_{2}=1$ lies in $D_{00}^{⊥}$, but not in $D_{00}$. So, $D_{00}^{⊥}\neq D_{00}$ and hence, $D_{00}$ is not a Dirac structure.

**Question** **4.** *Given F=C(a,b) and $E=C^{1}\left(a,b\right)$. Is*

$$D_{p}=\left\{\left(\begin{array}{c} f\\ e \end{array}\right)\in F\times E|f=\frac{de}{d\zeta },e\left(a\right)=e\left(b\right)\right\}$$

*a Dirac structure?*

**Answer**: Calculating $D_{p}^{⊥}$ leads to (see previous answer) that the following equalities have to hold for all $e\in C^{1}\left(a,b\right)$:

$$\begin{array}{cc} 0= & \;\int_{a}^{b}\left[f_{2}\left(\zeta \right)-\frac{de_{2}}{d\zeta }\left(\zeta \right)\right]e\left(\zeta \right)d\zeta +e_{2}\left(b\right)e\left(b\right)-e_{2}\left(a\right)e\left(a\right)\\ = & \;\int_{a}^{b}\left[f_{2}\left(\zeta \right)-\frac{de_{2}}{d\zeta }\left(\zeta \right)\right]e\left(\zeta \right)d\zeta +\left[e_{2}\left(b\right)-e_{2}\left(a\right)\right]e\left(a\right). \end{array}$$

Thus, $f_{2}\left(\zeta \right)=\frac{de_{2}}{d\zeta }\left(\zeta \right)$ and $e_{2}\left(b\right)=e_{2}\left(a\right)$. So $D_{p}^{⊥}=D_{p}$.

### 2.6. Dirac Structures, Link to Partial Differential Equations

In Question 4, we have seen that $D_{p}=\left\{\left(\begin{array}{c} f\\ e \end{array}\right)\in F\times E|f=\frac{de}{d\zeta },e\left(a\right)=e\left(b\right)\right\}$ is a Dirac structure. As we did in the finite-dimensional case, we can link a differential equation to it, by choosing $f=\dot{x}\left(t\right)$ and e=Hx(t).

Since *e* and *f* depend on ζ and thus x(t) depends on ζ as well, we now have to write $f=\frac{\partial x}{\partial t}$. $\left(\begin{array}{c} f\\ e \end{array}\right)\in D_{p}$ which is the same as $e\in C^{1}\left(a,b\right)$, $f=\frac{\partial e}{\partial \zeta }$ & e(b)=e(a) is now the same as writing $H\left(\cdot \right)x\left(\cdot ,t\right)\in C^{1}\left(a,b\right)$,

(21) $$\frac{\partial x}{\partial t}\left(\zeta ,t\right)=\frac{\partial }{\partial \zeta }\left[H(\zeta )x(\zeta ,t)\right]\;and\;H\left(a\right)x\left(a,t\right)=H\left(b\right)x\left(b,t\right).$$

So, a *Partial Differential Equation (PDE)* with *Boundary Conditions*.

As before, see e.g., Example 1, the Dirac structure gives that along solutions we have that $H\left(t\right)=\frac{1}{2}\int_{a}^{b}x{\left(\zeta ,t\right)}^{⊤}H\left(\zeta \right)x\left(\zeta ,t\right)d\zeta =\frac{1}{2}\int_{a}^{b}H\left(\zeta \right)x{\left(\zeta ,t\right)}^{2}d\zeta$ is constant.

Again, we see that the Dirac structure implies properties of the solution of a differential equation, but do we have solutions?

**Example** **6.** *We consider the PDE (21) with H=1, i.e.,*

$$\frac{\partial x}{\partial t}\left(\zeta ,t\right)=\frac{\partial x}{\partial \zeta }\left(\zeta ,t\right),\;x\left(a,t\right)=x\left(b,t\right).$$

*We impose the initial condition $x\left(\zeta ,0\right)=x_{0}\left(\zeta \right)$ with $x_{0}\in C^{1}\left(a,b\right)$ satisfying $x_{0}\left(a\right)=x_{0}\left(b\right)$, i.e., $x_{0}\in E$. The solution of this PDE is*

$$x\left(\zeta ,t\right)=x_{0,ext}\left(t+\zeta \right)$$

*with $x_{0,ext}$ the periodic extension of $x_{0}$, that is*

$$x_{0,ext}\left(\eta \right)=\left\{\begin{array}{cc} x_{0}\left(\eta \right) & \eta \in [a,b)\\ x_{0}\left(\eta +k\left(b-a\right)\right) & k\in Zs.t.\;\eta +k(b-a)\in [a,b). \end{array}\right.$$

**Figure 4:**
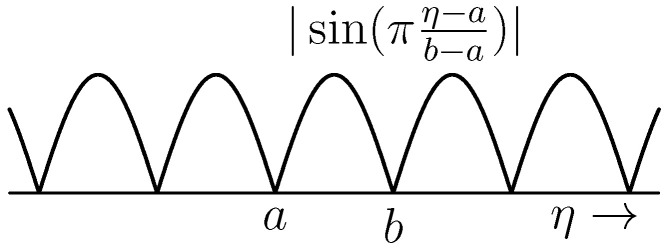
Periodic extension of $x_{0}\left(\eta \right)=\sin\left(\pi \frac{\eta -a}{b-a}\right)$.

*As it is clear from Figure 4, even when $x_{0}\in E=C^{1}\left(a,b\right)$ this does not imply that x(t,·)∈E for all t. Once more, we see that a Dirac structure does *not *guarantee the existence of solutions (in E).*

### 2.7. Dirac Structures, $P_{1},P_{0}$ Class

In the previous subsection, we studied Dirac structures for scalar functions, but we can easily extend it to vector-valued functions. The bilinear product (18) becomes $f\left(\zeta \right),e\left(\zeta \right)\in R^{n}$, ζ∈[a,b]

(22) $$\langle f|e\rangle =\int_{a}^{b}f{\left(\zeta \right)}^{⊤}e\left(\zeta \right)d\zeta .$$

The scalar relation, $f=\frac{de}{d\zeta }$, see, e.g., Questions 3 and 4 is replaced by

(23) $$f=P_{1}\frac{de}{d\zeta }+P_{0}e\;with\;P_{0},P_{1}\in R^{n\times n},P_{1}^{⊤}=P_{1},P_{0}^{⊤}=-P_{0}.$$

Next, we derive two fundamental relations involving this equation, (Theorem 3.1 [6]).

**Lemma** **4.** *For $e\in C^{1}\left(\left[a,b\right];R^{n}\right)$, $P_{1}\in R^{n\times n}$ satisfying $P_{1}^{⊤}=P_{1}$ the following equality holds*

(24) $$\int_{a}^{b}{\left[P_{1}\frac{de}{d\zeta }\left(\zeta \right)\right]}^{⊤}e\left(\zeta \right)d\zeta =\frac{1}{2}\left[e{\left(b\right)}^{⊤}P_{1}e\left(b\right)-e{\left(a\right)}^{⊤}P_{1}e\left(a\right)\right].$$

From (24) we observe that to make $V=\{f=P_{1}\frac{de}{d\zeta }\}$ into a Dirac structure w.r.t. the bilinear product (22), we have to add boundary conditions.

Therefore, we define *boundary flow* and *effort*

(25) $$\left(\begin{array}{c} f_{\partial }\\ e_{\partial } \end{array}\right)=\underset{R_{0}}{\underbrace{\frac{1}{\sqrt{2}}\left(\begin{array}{cc} P_{1} & -P_{1}\\ I & I \end{array}\right)}}\left(\begin{array}{c} e(b)\\ e(a) \end{array}\right).$$

With this we can extend and reformulate the equality (24).

For $e\in C^{1}\left(\left[a,b\right];R^{n}\right)$, $f=P_{1}\frac{de}{d\zeta }+P_{0}e$, with $P_{k}\in R^{n\times n}$, $P_{1}^{⊤}=P_{1}$, $P_{0}^{⊤}=-P_{0}$, we have that

(26) $$\langle f|e\rangle -f_{\partial }^{⊤}e_{\partial }=0.$$

**Exercise** **7.** *Prove Lemma 4 and the equality (26).*

**Exercise** **8.** *For $F=C(\left[a,b\right];R^{n})$ and $E=C^{1}\left(\left[a,b\right];R^{n}\right)$ define a Dirac structure around (23). Furthermore, prove that your candidate Dirac structure is a Dirac structure.*

*Hint: see Question 4.*

**Exercise** **9.** *For $F=C\left(\left[a,b\right];R^{n}\right)\times R^{n}$ and $E=C^{1}\left(\left[a,b\right];R^{n}\right)\times R^{n}$ define a Dirac structure around $f=P_{1}\frac{de}{d\zeta }+P_{0}e$ and (26). Furthermore, prove that your candidate Dirac structure is a Dirac structure.*

Similar to in Section 2.6, we can associate a PDE to these Dirac structures. More specifically to the relation between *f* and *e* as given in (23). This gives that $H\left(\cdot \right)x\left(\cdot ,t\right)\in C^{1}\left(\left[a,b\right];R^{n}\right)$,

(27) $$\frac{\partial x}{\partial t}\left(\zeta ,t\right)=P_{1}\frac{\partial }{\partial \zeta }\left[H(\zeta )x(\zeta ,t)\right]+P_{0}H\left(\zeta \right)x\left(\zeta ,t\right)$$

with (in)homogeneous boundary conditions. Of course the existence problem which we found in the scalar case will still remain.

At this moment, it is unclear if the PDE in (27) is worth studying, therefore, we present a simple example next.

**Example** **7.** *In this example, we consider the PDE (27) for n=2, $P_{0}=0$,*

(28) $$P_{1}=\left(\begin{array}{cc} 0 & 1\\ 1 & 0 \end{array}\right),\;H\left(\zeta \right)=\left(\begin{array}{cc} c & 0\\ 0 & c \end{array}\right).$$

*Note that $P_{0}$ and $P_{1}$ satisfy the conditions of (23). Substituting the matrices $P_{1}$, $P_{0}$, and H into (27), we find*

$$\frac{\partial }{\partial t}\left(\begin{array}{c} x_{1}\\ x_{2} \end{array}\right)=\frac{\partial x}{\partial t}=\left(\begin{array}{cc} 0 & 1\\ 1 & 0 \end{array}\right)\frac{\partial }{\partial \zeta }\left[\left(\begin{array}{cc} c & 0\\ 0 & c \end{array}\right)x\right]=\frac{\partial }{\partial \zeta }\left(\begin{array}{c} cx_{2}\\ cx_{1} \end{array}\right).$$

*For $x_{1}$ this becomes*

$$\frac{\partial^{2}x_{1}}{\partial t^{2}}=\frac{\partial }{\partial t}\left[\frac{\partial x_{1}}{\partial t}\right]=\frac{\partial }{\partial t}\left[\frac{\partial cx_{2}}{\partial \zeta }\right]=c\frac{\partial }{\partial \zeta }\left[\frac{\partial x_{2}}{\partial t}\right]=c^{2}\frac{\partial^{2}x_{1}}{\partial \zeta^{2}}.$$

*In this PDE we recognise the *wave equation.

*Alternatively, when we are given the wave equation*

$$\frac{\partial^{2}w}{\partial t^{2}}\left(\zeta ,t\right)=c^{2}\frac{\partial^{2}w}{\partial \zeta^{2}}\left(\zeta ,t\right),$$

*then by defining $x_{1}=c^{-1}\frac{\partial w}{\partial t}$, and $x_{2}=\frac{\partial w}{\partial \zeta }$ we find*

$$\frac{\partial }{\partial t}\left(\begin{array}{c} x_{1}\\ x_{2} \end{array}\right)=\frac{\partial }{\partial \zeta }\left(\begin{array}{c} cx_{2}\\ cx_{1} \end{array}\right)=\left(\begin{array}{cc} 0 & 1\\ 1 & 0 \end{array}\right)\frac{\partial }{\partial \zeta }\left[\left(\begin{array}{cc} c & 0\\ 0 & c \end{array}\right)x\right].$$

*This is (27) with $P_{0}=0$ and $P_{1}$, H as in (28).*

The above example shows that the PDE (27) encodes interesting cases.

### 2.8. Dirac Structures, Higher Spatial Dimension

We have only discussed (potential) infinite-dimensional Dirac structures in one spatial variable. However, there is no fundamental reason for that. In fact, in [5] infinite-dimensional Dirac structures are introduced using differential forms, imposing no conditions on the spatial dimension. Similar to their Example 3.4, consider the following relation

$$\left(\begin{array}{c} f_{1}\\ f_{2} \end{array}\right)=\left(\begin{array}{cc} 0 & \mathrm{div}\\ \mathrm{grad} & 0 \end{array}\right)\left(\begin{array}{c} e_{1}\\ e_{2} \end{array}\right)$$

on the set $\Omega \subset R^{3}$ with a smooth boundary. We assume that $e_{1}\in C^{1}\left(\Omega ;R\right)$, $e_{2}\in C^{1}\left(\Omega ;R^{3}\right)$, $f_{1}\in C\left(\Omega ;R\right)$, $f_{2}\in C\left(\Omega ;R^{3}\right)$. As bilinear product we take, see (22),

$$\langle f|e\rangle =\int_{\Omega }\left[f_{1}e_{1}+f_{2}^{⊤}e_{2}\right]d\omega .$$

Substituting the expression for $f_{1}$ and $f_{2}$, we find by standard theorem in vector calculus that

$$\begin{array}{cc} \langle f|e\rangle = & \;\int_{\Omega }\left[e_{1}\mathrm{div}\left(e_{2}\right)+e_{2}^{⊤}\mathrm{grad}\left(e_{1}\right)\right]d\omega \\ = & \int_{\Omega }\mathrm{div}\left(e_{1}e_{2}\right)d\omega =\int_{\Gamma }e_{1}e_{2}\cdot n\;d\eta , \end{array}$$

where Γ is the boundary of Ω and n is the outward unit normal. This is the higher spatial analogy of (24) and can serve as the starting point for defining Dirac structures, see e.g., [13].

### 2.9. Looking Back and Looking Forward

We have introduced Dirac structures and shown how, in a finite-dimensional bond space, they can be characterised. Furthermore, we showed that ordinary differential equations defined on it have a conserved quantity, which is encoded in the Dirac structure. When the bond space is infinite-dimensional, i.e., given by the product of two function spaces, we can still define Dirac structures. Furthermore, we can link these infinite-dimensional Dirac structures to (*partial*) differential equations. However, we have trouble (even in simple cases) obtaining the existence of solutions for these PDEs.

To solve this matter we take a more abstract/functional analytic point of view.

### 2.10. Dirac Structure, Operators

For finite-dimensional spaces, we had that {f=Je} defines a Dirac structure if and only if $J^{⊤}=-J$, see Lemma 1. So a natural question is whether a similar result holds for infinite-dimensional spaces. To answer this question, we have to introduce some notions from operator theory/functional analysis.

We let *X* be a real *Hilbert space* with *inner product*〈·,·〉.

**Definition** **2.** *Let Q:dom(Q)⊆X↦X be a densely defined linear operator. The *adjoint*, $Q^{*}$, of Q is defined as follows*

$$\mathrm{dom}\left(Q^{*}\right)=\left\{z\in X|\exists w\in X\;s.t.\;\langle Qx,z\rangle =\langle x,w\rangle ,\forall x\in \mathrm{dom}\left(Q\right)\right\}.$$

*For $z\in \mathrm{dom}(Q^{*})$, we define $Q^{*}\left(z\right)=w$.*

So, as in finite-dimensions, the adjoint, $Q^{*}$, of *Q* satisfies

$$\langle Qx,z\rangle =\langle x,Q^{*}z\rangle ,\;x\in \mathrm{dom}\left(Q\right),z\in \mathrm{dom}\left(Q^{*}\right)$$

Often the hard part of finding the adjoint is to determine its domain.

**Definition** **3.** *Let Q:dom(Q)⊆X↦X be a densely defined linear operator.*

- *Q is skew-adjoint when $Q^{*}=-Q$.*
- *Q is self-adjoint when $Q^{*}=Q$.*

Note that these definitions imply that $\mathrm{dom}\left(Q^{*}\right)=\mathrm{dom}\left(Q\right)$.

**Exercise** **10.** *Given F=E=X, with X a Hilbert space with inner product ${\langle f,e\rangle }_{X}$. Show that the following defines a non-degenerate bilinear product on F×E*

(29) $$\langle f|e\rangle ={\langle f,e\rangle }_{X}.$$

The following theorem gives a beautiful characterisation of Dirac structures in Hilbert spaces and can be seen as a generalisation of Lemma 1.

**Theorem** **1.** *Let F=E=X, with X a Hilbert space and J:dom(J)⊆X↦X. Then,*

(30) $$D=\left\{\left(\begin{array}{c} f\\ e \end{array}\right)\in F\times E|f=Je,e\in \mathrm{dom}\left(J\right)\right\}$$

*is a Dirac structure w.r.t. the bilinear product (29) if and only if J is skew-adjoint.*

**Proof.**  We will calculate $D^{⊥}$. By definition $\left(\begin{array}{c} f_{2}\\ e_{2} \end{array}\right)\in D^{⊥}$ if and only if, see (5),

$$\langle \left(\begin{array}{c} f_{2}\\ e_{2} \end{array}\right)|\left(\begin{array}{c} f\\ e \end{array}\right)\rangle =0\;\forall \left(\begin{array}{c} f\\ e \end{array}\right)\in D.$$

Using the definition of the bilinear product, (29), and that of the set D, (30), this equation becomes

$${\langle f_{2},e\rangle }_{X}+{\langle Je,e_{2}\rangle }_{X}=0\;\forall e\in \mathrm{dom}\left(J\right),$$

or equivalently,

$${\langle Je,e_{2}\rangle }_{X}=-{\langle f_{2},e\rangle }_{X}=-{\langle e,f_{2}\rangle }_{X}\;\forall e\in \mathrm{dom}\left(J\right).$$

Definition 2 implies that $e_{2}\in \mathrm{dom}\left(J^{*}\right)$ and $J^{*}e_{2}=-f_{2}$.

Summarising, we have determined $D^{⊥}$ as

(31) $$D^{⊥}=\left\{\left(\begin{array}{c} f_{2}\\ e_{2} \end{array}\right)\in F\times E|f_{2}=-J^{*}e_{2},e_{2}\in \mathrm{dom}\left(J^{*}\right)\right\}.$$

Now, if D is a Dirac structure, then by (30) and (31) $\mathrm{dom}\left(J^{*}\right)=\mathrm{dom}\left(J\right)$ and $-J^{*}=J$. In other words, *J* is skew-adjoint. Alternatively, if *J* is skew-adjoint, then this implies that the sets of (30) and (31) are the same, and thus D is a Dirac structure. □

### 2.11. Dirac Structures, Revisited

In Question 4, we have shown that

(32) $$D_{p}=\left\{\left(\begin{array}{c} f\\ e \end{array}\right)\in C\left(a,b\right)\times C^{1}\left(a,b\right)|f=\frac{de}{d\zeta },e\left(a\right)=e\left(b\right)\right\}$$

is a Dirac structure. However, this does not fit into Theorem 1 for the simple reasons that F≠E and neither of them is a Hilbert space.

However, $f=\frac{de}{d\zeta }$ in (32) looks very similar to f=Je in (30). Furthermore, the bilinear product (18) used in Question 4 looks very similar to an inner product. So it is logical to hope that we can change the definition of $D_{p}$ slightly such that it fits into that Theorem 1. Indeed, this is possible.

Namely, we take

- $F=E=L^{2}\left(a,b\right)$ all measurable, square integrable, real-valued, scalar functions on the interval (a,b);
- $Je=\frac{de}{d\zeta }$ with dom$\left(J\right)=\{e\in H^{1}\left(a,b\right)|e\left(a\right)=e\left(b\right)\}$. Here, $H^{1}\left(a,b\right)$ are all functions in $L^{2}\left(a,b\right)$ that have a derivative in $L^{2}\left(a,b\right)$.

Then, *J* is skew-adjoint, and thus,

(33) $$\begin{array}{cc} D_{per}= & \;\left\{\left(\begin{array}{c} f\\ e \end{array}\right)\in L^{2}\left(a,b\right)\times L^{2}\left(a,b\right)|e\in \mathrm{dom}\left(J\right),f=Je\right\}\\ = & \;\left\{\left(\begin{array}{c} f\\ e \end{array}\right)\in L^{2}\left(a,b\right)\times H^{1}\left(a,b\right)|f=\frac{de}{d\zeta },e\left(a\right)=e\left(b\right)\right\} \end{array}$$

is a Dirac structure. The proof that *J* is skew-adjoint is not simple and is based on the following result. Given $f_{2},e_{2}\in L^{2}\left(a,b\right)$, if for every $e\in H^{1}\left(a,b\right)$ satisfying e(a)=0=e(b) the following holds

$$\int_{a}^{b}\frac{de}{d\zeta }\left(\zeta \right)f_{2}\left(\zeta \right)d\zeta =\int_{a}^{b}e\left(\zeta \right)e_{2}\left(\zeta \right)d\zeta ,$$

then, $f_{2}\in H^{1}\left(a,b\right)$ and ${\dot{f}}_{2}=-e_{2}$. We will return to Dirac structures like (33) in Section 3.

### 2.12. Dirac Structures, from Infinite- to Finite-Dimensional

In this subsection, we construct a finite-dimensional Dirac structure from an infinite-dimensional one. Since infinite-dimensional Dirac structures are often related to PDEs with a conserved physical quantity, like energy, it is desirable that a (numerical) approximation maintains this quantity. By approximating the Dirac structure, this is guaranteed. We will only show one option, many variations on this idea exist.

Given the infinite-dimensional Dirac structure of the form, see e.g., (33) or Question 4,

$$D_{\infty }=\left\{\left(\begin{array}{c} f\\ e \end{array}\right)\in F\times E|f=Je\right\}$$

we can easily obtain a *finite*-dimensional Dirac structure. Therefore, we choose $e_{1},\cdots ,e_{N}$ (independent) elements of E, and define $f_{k}=Je_{k}$, k=1,⋯N. Next, we define

- $E_{N}:=\mathrm{span}\left\{e_{1},\cdots ,e_{N}\right\}\subset E$;
- $F_{N}:=\mathrm{span}\left\{f_{1},\cdots ,f_{N}\right\}\subset F$;
- For $\left(f,e\right)\in F_{N}\times E_{N}$ the bilinear product is defined as ${\langle f|e\rangle }_{N}:=\langle f|e\rangle$.
-
$D_{N}:=\left\{\left(\begin{array}{c} f\\ e \end{array}\right)\in F_{N}\times E_{N}|f=Je\right\}\subset D$

**Exercise** **11.** *Prove that $D_{N}$ is a Dirac structure in $F_{N}\times E_{N}$ if and only if $\dim F_{N}=N$.*

We illustrate the above construction in more detail for the Dirac structure (33). To simplify notation, we take a=0 and b=1. So, we are given the Dirac structure

$$D_{per}=\left\{\left(\begin{array}{c} f\\ e \end{array}\right)\in L^{2}\left(0,1\right)\times H^{1}\left(0,1\right)|f=\frac{de}{d\zeta },e\left(0\right)=e\left(1\right)\right\}.$$

We choose N∈N and define $h=N^{-1}$. Furthermore, let $\zeta_{k}:=k\cdot h$, k=0,1,⋯,N. With this we define for k=1,⋯,N−1 “hat” functions in E as

$$e_{k}\left(\zeta \right)=\left\{\begin{array}{cc} N(\zeta -\zeta_{k-1}) & \zeta \in [\zeta_{k-1},\zeta_{k}];\\ N(\zeta_{k+1}-\zeta ) & \zeta \in [\zeta_{k},\zeta_{k+1}];\\ 0 & elsewhere. \end{array}\right.$$

and

$$e_{N}\left(\zeta \right)=\left\{\begin{array}{cc} N(\zeta -\zeta_{N-1}) & \zeta \in [\zeta_{N-1},\zeta_{N}];\\ N(\zeta_{1}-\zeta ) & \zeta \in [0,h];\\ 0 & elsewhere, \end{array}\right.$$

see also Figure 5.

**Figure 6:**
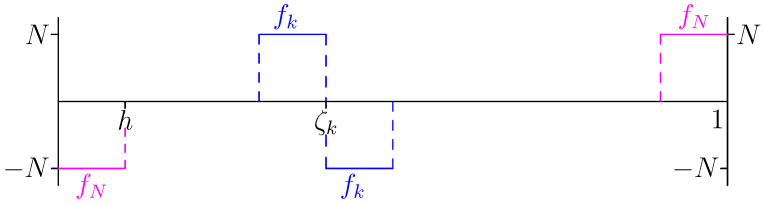
The step-functions, $f_{k}$.

From $f_{k}=Je_{k}=\frac{de_{k}}{d\zeta }$, we find, see also Figure 6,

$$f_{k}\left(\zeta \right)=\left\{\begin{array}{cc} N & \zeta \in (\zeta_{k-1},\zeta_{k});\\ -N & \zeta \in (\zeta_{k},\zeta_{k+1});\\ 0 & elsewhere, \end{array}\right.\;f_{N}\left(\zeta \right)=\left\{\begin{array}{cc} N & \zeta \in (\zeta_{N-1},\zeta_{N});\\ -N & \zeta \in (0,h);\\ 0 & elsewhere. \end{array}\right.$$

It is easy to show that $\dim\left({\mathrm{span}}_{k=1,\cdots ,N}\left\{f_{k}\right\}\right)=N$, and thus, by Exercise 11, $D_{N}=\left\{\left(\begin{array}{c} f\\ e \end{array}\right)\in F_{N}\times E_{N}|f=Je\right\}$ is a Dirac structure (finite-dimensional).

Since $\dim E_{N}=\dim F_{N}=N$, we can define an equivalent Dirac structure on $R^{N}\times R^{N}$.

For $e\in E_{N}$ and $f\in F_{N}$ given as $e\left(\zeta \right)=\sum_{k=1}^{N}a_{k}e_{k}\left(\zeta \right)$ and $f\left(\zeta \right)=\sum_{k=1}^{N}b_{k}f_{k}\left(\zeta \right)$, respectively, we define

$$\vec{e}=\left(\begin{array}{c} a_{1}\\ \vdots \\ a_{N} \end{array}\right),\;\vec{f}=\left(\begin{array}{c} b_{1}\\ \vdots \\ b_{N} \end{array}\right).$$

By definition $f_{k}=Je_{k}$. Thus, the Dirac structure, becomes

(34) $$D_{N}=\left\{\left(\begin{array}{c} \vec{f}\\ \vec{e} \end{array}\right)\in R^{N}\times R^{N}|\vec{f}=\vec{e}\right\}.$$

**Question** **5.** *What do you find strange and or weird in the $D_{N}$ as defined in (34)?*

We take a closer look at the bilinear product between $\vec{f}$ and $\vec{e}$. A straightforward calculation gives

〈fk∣eℓ〉=∫01fk(ζ)ek(ζ)dζ=−12N2k=ℓ+1−12N2k=ℓ−1−120elsewhere

In particular, $\langle f_{k}|e_{k}\rangle =0$, and thus,

$$\langle f|e\rangle \neq {\vec{f}}^{⊤}\vec{e}.$$

Instead of this, we find

$$\langle f|e\rangle ={\vec{f}}^{⊤}Q\vec{e}$$

with $Q_{k,l}=\langle f_{k}|e_{\ell }\rangle$. (see also Exercise 1)

What we have shown above can serve as a starting point for *structure preserving* numerical discretisation of partial differential equations. It is close to the work of [14], or more recently [15]. Other approaches have been developed as well, see for instance [16] in which the authors use a dedicated version of the mixed finite element method for obtaining a structure preserving discretisation.

## 3. Abstract Differential Equations

### 3.1. Introduction and Notation

In this part, we go into existence theory for *linear* PDEs. We will focus on those on a *one-dimensional* spatial domain, and will study homogeneous and inhomogeneous equations. After studying the existence and uniqueness, we will come back to the Dirac structure of the previous section, and discuss which properties of the PDE are a consequence of the Dirac structure.

Some notation:

- In this part, we denote the one-dimensional spatial domain by [0,ℓ]. Hence, we shifted the interval [a,b] by *a* and denoted b−a by *ℓ*. However, we have kept units (which will be invisible when choosing the interval [0,1]).
- On the Hilbert space *X* the norm is denoted by ∥·∥ and the inner product by 〈·,·〉.

### 3.2. Solutions of PDEs

To introduce and motivate solutions of a PDE, we consider the following simple PDE with ζ∈[0,ℓ] and t≥0

(35) $$\frac{\partial w}{\partial t}\left(\zeta ,t\right)=\frac{\partial w}{\partial \zeta }\left(\zeta ,t\right),\;w\left(\ell ,t\right)=0,\;w\left(\zeta ,0\right)=w_{0}\left(\zeta \right).$$

We call a function w:[0,ℓ]×[0,∞)→R a *classical solution* of (35), if *w* is continuously differentiable, and for all t≥0, ζ∈[0,ℓ] the differential equation, initial, and boundary conditions in (35) are satisfied.

**Exercise** **12.** *Determine the classical solution of (35) for $w_{0}\left(\zeta \right)=\sin\left(\pi \zeta /\ell \right)$.*

History has shown that the concept of a classical solution is too restrictive, and that a weaker concept of a solution was needed. We illustrate this for the PDE (35).

We take a smooth *test function* ϕ(ζ) and integrate over the spatial domain the PDE of (35).

$$\begin{array}{cc} \int_{0}^{\ell }\phi \left(\zeta \right)\frac{\partial w}{\partial t}\left(\zeta ,t\right)d\zeta = & \;\int_{0}^{\ell }\phi \left(\zeta \right)\frac{\partial w}{\partial \zeta }\left(\zeta ,t\right)d\zeta \;\left(PDE\right)\\ \left(int.\;by\;parts\right)\;= & \;{\left[\phi (\zeta )w(\zeta ,t)\right]}_{0}^{\ell }-\int_{0}^{\ell }\dot{\phi }\left(\zeta \right)w\left(\zeta ,t\right)d\zeta \\ \left(b.c.\right)\;= & \;-\phi \left(0\right)w\left(0,t\right)-\int_{0}^{\ell }\dot{\phi }\left(\zeta \right)w\left(\zeta ,t\right)d\zeta . \end{array}$$

If we take test functions satisfying ϕ(0)=0, we find

$$\frac{d}{dt}\int_{0}^{\ell }\phi \left(\zeta \right)w\left(\zeta ,t\right)d\zeta =\int_{0}^{\ell }\phi \left(\zeta \right)\frac{\partial w}{\partial t}\left(\zeta ,t\right)d\zeta =-\int_{0}^{\ell }\dot{\phi }\left(\zeta \right)w\left(\zeta ,t\right)d\zeta .$$

Integrating this expression with respect to time from t=0 to $t=t_{f}$ gives

(36) $$\int_{0}^{\ell }\phi \left(\zeta \right)w\left(\zeta ,t_{f}\right)d\zeta -\int_{0}^{\ell }\phi \left(\zeta \right)w\left(\zeta ,0\right)d\zeta =-\int_{0}^{t_{f}}\int_{0}^{\ell }\dot{\phi }\left(\zeta \right)w\left(\zeta ,t\right)d\zeta .$$

You see that there are *no derivatives* of *w* taken anymore.

Now, we call w(ζ,t) a *weak* or *mild solution* of the PDE (35) if Equation (36) is satisfied for all smooth test functions ϕ satisfying ϕ(0)=0.

It is easy to see that a classical solution is always a weak solution, but the converse need not to be held.

Note that we have not specified the set of initial conditions yet. There is some freedom in this. For instance, if we are interested in a positive solution, i.e., w(ζ,t) is non-negative at each position and time, then we can choose that as our set of initial conditions. However, for the existence theory of PDEs, it is more common to choose a Hilbert or Banach space of functions, see also Appendix B. We choose a Hilbert space, which we denote by *X*. Thus, by choosing this, we not only have chosen the set of initial conditions, but also the set in which our solution at time *t* will lie.

**Exercise** **13.** *For a given $w_{0}\in X=L^{2}\left(0,\ell \right)$ show that*

$$w\left(\zeta ,t\right)=\left\{\begin{array}{cc} w_{0}\left(\zeta +t\right) & \zeta +t\in [0,\ell ]\\ 0 & elsewhere \end{array}\right.$$

*is the weak solution of*

$$\frac{\partial w}{\partial t}\left(\zeta ,t\right)=\frac{\partial w}{\partial \zeta }\left(\zeta ,t\right),\;w\left(\ell ,t\right)=0,\;w\left(\zeta ,0\right)=w_{0}\left(\zeta \right).$$

We will now study when our PDE has a weak solution.

Note there is a difference between knowing the existence of a solution and having the form/expression of the solution. The expression for the solution can be hard/impossible to find. So we concentrate on *existence*. We will use the abstract formulation as shortly summarised in Appendix B. We concentrate on contraction semigroups, i.e., on solutions satisfying the additional property that

$$∥x\left(t\right)∥\leq ∥x_{0}∥\;\forall t>0\;\left(contraction\right),$$

where ∥·∥ denotes the norm of the *state space X* and $x_{0}$ is the initial condition, i.e., $x\left(0\right)=x_{0}$. In general terms this means that the solution stays always less than the initial value.

Since our PDEs are linear, the above inequality implies that any two solutions satisfy

$$∥x_{1}\left(t\right)-x_{2}\left(t\right)∥\;\leq \;∥x_{10}-x_{20}∥,\;t\geq 0.$$

This can be interpreted in two ways. First, it gives that if two solutions start close together, then they will stay close together. Alternatively, it can be regarded as that solutions depend continuously on the initial condition.

### 3.3. Abstract Differential Equations and Dirac Structures

From now on, we assume that *X* is a Hilbert space with inner product 〈·,·〉.

From Theorem 1, we have that if J:dom(J)⊆X↦X is skew-adjoint, then,

$$D=\left\{\left(\begin{array}{c} f\\ e \end{array}\right)\in F\times E|f=Je,e\in \mathrm{dom}\left(J\right)\right\}$$

is a Dirac structure on B=F×E=X×X.

By Theorem A1, we have that this skew-adjoint operator *J* generates a $C_{0}$-semigroup (unitary group) on the Hilbert space *X*, i.e, the abstract differential equation (ADE)

(37) $$\dot{x}\left(t\right)=Jx\left(t\right),\;x\left(0\right)=x_{0}$$

has for every initial condition, $x_{0}$ in *X*, a unique solution x(t). Furthermore, this solution satisfies $∥x\left(t\right)∥\;=\;∥x_{0}∥$, t≥0.

As is clear from the finite-dimensional case, see, e.g., Example 1, we do not want to look at differential equations of the type (37), but more often of the type

(38) $$\dot{x}\left(t\right)=JHx\left(t\right),\;x\left(0\right)=x_{0}.$$

That is, we have connected to the Dirac structure a storage, see Figure 3 and Figure 7.

**Question** **6.** *Does the abstract differential Equation (38) possess a (unique) solution for all $x_{0}\in X$?*

Based on Lemma A1 we answer this question positively, but we need that mI≤H≤MI for some m,M>0.

So we see that the skew-adjointness of the defining operator in the Dirac structure gives you also uniqueness and existence of solutions to the corresponding (abstract) differential equation. Note that the Dirac structure gives you that $H\left(t\right):=\frac{1}{2}\langle x\left(t\right),Hx\left(t\right)\rangle$. If H(t) has the meaning “*energy*”, then we can phrase this as; the solution exists for every initial condition with finite energy, and the energy stays constant along the solution.

Since normally differential equations are not given in an abstract form, but appear as ordinary or partial differential equations, we study partial differential equations defined via a Dirac structure in the following section.

### 3.4. Partial Differential Equations and Dirac Structures

We study PDEs related to the class of operators introduced in Section 2.7. That is, we study the class of PDEs on the spatial interval [0,ℓ] given as

$$\frac{\partial x}{\partial t}\left(\zeta ,t\right)=P_{1}\frac{\partial }{\partial \zeta }\left[H(\zeta )x(\zeta ,t)\right]+P_{0}H\left(\zeta \right)x\left(\zeta ,t\right),$$

with $x\left(\zeta ,t\right)\in R^{n}$, $P_{1},P_{0}\in R^{n\times n}$ satisfying $P_{1}^{⊤}=P_{1}$ and $P_{0}=-P_{0}^{⊤}$. Furthermore, we assume that $P_{1}$ is invertible.

In Section 2.7 we studied Dirac structures on $F\times E=C\left(\left[0,\ell \right];R^{n}\right)\times C^{1}\left(\left[0,\ell \right];R^{n}\right)$ and on $F\times E=\left(C\left(\left[0,\ell \right];R^{n}\right)\times R^{n}\right)\times \left(C^{1}\left(\left[0,\ell \right];R^{n}\right)\times R^{n}\right)$ associated to the above PDE, see Exercises 8 and 9. So the F and E were different. However, to apply the theory of abstract differential equations, we need the flow and effort space to be the same. Since we still may choose the domain of *J*, we can make sure that the derivative (with respect to ζ) exists.

We choose $X=F=E=L^{2}\left(\left(0,\ell \right);R^{n}\right)\times R^{n}$ and as bilinear product $\langle \left(\begin{array}{c} f\\ f_{\partial } \end{array}\right)|(\begin{array}{c} e\\ e_{\partial } \end{array})\rangle =\langle f,e\rangle -f_{\partial }^{⊤}e_{\partial }$. The (candidate) Dirac structure on X×X then becomes, see also Figure 8,

(39) $$\begin{array}{cc} D= & \;\left\{\left(\begin{array}{c} f\\ f_{\partial }\\ e\\ e_{\partial } \end{array}\right)\in L^{2}\left(\left(0,\ell \right);R^{n}\right)\times R^{n}\times L^{2}\left(\left(0,\ell \right);R^{n}\right)\times R^{n}|\right.\\ \; & \;\;\;e\in H^{1}\left(\left(0,\ell \right);R^{n}\right),f=P_{1}\frac{de}{d\zeta }+P_{0}e,\\ \; & \;\;\;\left.\left(\begin{array}{c} f_{\partial }\\ e_{\partial } \end{array}\right)=\underset{R_{0}}{\underbrace{\frac{1}{\sqrt{2}}\left(\begin{array}{cc} P_{1} & -P_{1}\\ I & I \end{array}\right)}}\left(\begin{array}{c} e(\ell )\\ e(0) \end{array}\right)\right\}. \end{array}$$

Here, $H^{1}\left(\left(0,\ell \right);R^{n}\right)$ is the (Sobolev) space of all functions from (0,ℓ) to $R^{n}$ whose derivative lies in $L^{2}\left(\left(0,\ell \right);R^{n}\right)$.

It is not hard to show that D of (39) satisfies $D\subseteq D^{⊥}$, see also (26). The other inclusion also holds, but is much harder to show. It uses the precise definition of $H^{1}$. If in (39) we were to replace *e* by x(·,t) and *f* by $\frac{\partial x}{\partial t}$, then we obtain the PDE stated in the beginning of this section, but since $f_{\partial }$ and $e_{\partial }$, and thus the boundary conditions, are not specified, we will not have a unique solution. To solve this problem, we have to specify $f_{\partial }$ and $e_{\partial }$. We do this via a second Dirac structure $D_{bc}\subset R^{n}\times R^{n}$, and letting the boundary flow and effort lie in this Dirac structure. Thus, if the $D_{bc}$ is given via the range of the (full rank) matrix $\left(\begin{array}{c} F\\ E \end{array}\right)$ with $E^{⊤}F+F^{⊤}E=0$ (see Lemma 1), then the Dirac structure in the variables *f* and *e* becomes, see also (39),

(40) $$\begin{array}{cc} D\circ D_{bc}= & \;\left\{\left(\begin{array}{c} f\\ e \end{array}\right)\in L^{2}\left(\left(0,\ell \right);R^{n}\right)\times L^{2}\left(\left(0,\ell \right);R^{n}\right)|e\in H^{1}\left(\left(0,\ell \right);R^{n}\right),\right.\\ \; & \;\;\;f=P_{1}\frac{de}{d\zeta }+P_{0}e,\left.R_{0}\left(\begin{array}{c} e(\ell )\\ e(0) \end{array}\right)\in \mathrm{ran}\left(\begin{array}{c} F\\ E \end{array}\right)\right\}. \end{array}$$

Based on Figure 8, the Dirac structure (40) is depicted in Figure 9.

Using the result of Theorem 1, we prove that this is a Dirac structure. The original proof can be found in [6], although this is for a larger class of differential operators.

**Theorem** **2** **([6]).** *The subspace $D\circ D_{bc}$ of Equation (40) with $P_{1},P_{0},F,E\in R^{n\times n}$, $P_{1}$ invertible and symmetric, $P_{0}$ skew-symmetric, and $\left(\begin{array}{c} F\\ E \end{array}\right)$ a full rank matrix with $E^{⊤}F+F^{⊤}E=0$ is a Dirac structure.*

**Proof.**  We begin by showing that $D\circ D_{bc}\subseteq {\left(D\circ D_{bc}\right)}^{⊥}$. Using Lemma 4, we have for $\left(\begin{array}{c} f\\ e \end{array}\right)\in D\circ D_{bc}$

$$\begin{array}{cc} \langle f|e\rangle = & \;\langle f,e\rangle =\frac{1}{2}\left[e{\left(\ell \right)}^{⊤}P_{1}e\left(\ell \right)-e{\left(0\right)}^{⊤}P_{1}e\left(0\right)\right]\\ = & \;\frac{1}{2}{\left(\begin{array}{c} e(\ell )\\ e(0) \end{array}\right)}^{⊤}\left(\begin{array}{cc} P_{1} & 0\\ 0 & -P_{1} \end{array}\right)\left(\begin{array}{c} e(\ell )\\ e(0) \end{array}\right)\\ = & \;\frac{1}{2}{\left(R_{0}^{-1}\left(\begin{array}{c} F\\ E \end{array}\right)z\right)}^{⊤}\left(\begin{array}{cc} P_{1} & 0\\ 0 & -P_{1} \end{array}\right)R_{0}^{-1}\left(\begin{array}{c} F\\ E \end{array}\right)z, \end{array}$$

with $z\in R^{n}$, see the last condition in (40). Since $R_{0}^{-1}=\frac{1}{\sqrt{2}}\left(\begin{array}{cc} P_{1}^{-1} & I\\ -P_{1}^{-1} & I \end{array}\right)$, we find

$$R_{0}^{-⊤}\left(\begin{array}{cc} P_{1} & 0\\ 0 & -P_{1} \end{array}\right)R_{0}^{-1}=\left(\begin{array}{cc} 0 & I\\ I & 0 \end{array}\right).$$

Combining this with the expression for 〈f|e〉 and the property of F,E, gives

$$\langle f|e\rangle =\frac{1}{2}z^{⊤}\left(F^{⊤}E+E^{⊤}F\right)z=0.$$

Hence, $D\circ D_{bc}\subseteq {\left(D\circ D_{bc}\right)}^{⊥}$.

Next, we show that ${\left(D\circ D_{bc}\right)}^{⊥}\subseteq D\circ D_{bc}$. Let $\left(\begin{array}{c} f_{1}\\ e_{1} \end{array}\right)\in L^{2}\left(\left(0,\ell \right);R^{n}\right)\times L^{2}\left(\left(0,\ell \right);R^{n}\right)$ be orthogonal to any $\left(\begin{array}{c} f\\ e \end{array}\right)\in D\circ D_{bc}$. From the strong overlap between the bilinear product and the bilinear product associated to the Dirac structure D of (39), we have that this gives

$$\left(\begin{array}{c} f_{1}\\ 0\\ e_{1}\\ 0 \end{array}\right)⊥D\Leftrightarrow \left(\begin{array}{c} f_{1}\\ 0\\ e_{1}\\ 0 \end{array}\right)\in D^{⊥}=D.$$

In particular, $e_{1}\in H^{1}\left(\left(0,\ell \right);R^{n}\right)$ and $f_{1}=P_{1}\frac{de_{1}}{d\zeta }+P_{0}e_{1}$.

Using the relation between *f* and *e* and between $f_{1}$ and $e_{1}$, plus integration by parts, we obtain

(41) $$\begin{array}{cc} 0= & \langle f_{1}|e\rangle +\langle f|e_{1}\rangle \\ = & \int_{0}^{\ell }{\left(P_{1}\frac{de_{1}}{d\zeta }\left(\zeta \right)+P_{0}e_{1}\left(\zeta \right)\right)}^{⊤}e\left(\zeta \right)+{\left(P_{1}\frac{de}{d\zeta }\left(\zeta \right)+P_{0}e\left(\zeta \right)\right)}^{⊤}e_{1}\left(\zeta \right)d\zeta \\ = & e_{1}{\left(\ell \right)}^{⊤}P_{1}e\left(\ell \right)-e_{1}{\left(0\right)}^{⊤}P_{1}e\left(0\right). \end{array}$$

Using the boundary conditions for *e*, see (40), this can be written as

(42) $$\begin{array}{cc} 0= & {\left(\begin{array}{c} e_{1}\left(\ell \right)\\ e_{1}\left(0\right) \end{array}\right)}^{⊤}\left(\begin{array}{cc} P_{1} & 0\\ 0 & -P_{1} \end{array}\right)R_{0}^{-1}\left(\begin{array}{c} F\\ E \end{array}\right)z\\ = & \frac{1}{\sqrt{2}}{\left(\begin{array}{c} e_{1}\left(\ell \right)\\ e_{1}\left(0\right) \end{array}\right)}^{⊤}\left(\begin{array}{cc} I & P_{1}\\ I & -P_{1} \end{array}\right)\left(\begin{array}{c} F\\ E \end{array}\right)z\\ = & \frac{1}{\sqrt{2}}{\left[\left(\begin{array}{cc} I & I\\ P_{1} & -P_{1} \end{array}\right)\left(\begin{array}{c} e_{1}\left(\ell \right)\\ e_{1}\left(0\right) \end{array}\right)\right]}^{⊤}\left(\begin{array}{c} F\\ E \end{array}\right)z. \end{array}$$

Since for any $z\in R^{n}$ we can find an $e\in H^{1}\left(\left(0,\ell \right);R^{n}\right)$ such that $\left(\begin{array}{c} f\\ e \end{array}\right)\in D\circ D_{bc}$, we see that (42) is satisfied for all *z*, i.e., all elements in the range $\left(\begin{array}{c} F\\ E \end{array}\right)$. This range equals the (finite-dimensional) Dirac structure $D_{bc}$.

An element $\left(\begin{array}{c} v\\ w \end{array}\right)$ lies in $D_{bc}^{⊥}$ if and only if $v^{⊤}Ez+w^{⊤}Fz=0$ for all $z\in R^{n}$, see (5). Combining this with (42), we see that $\left(\begin{array}{cc} P_{1} & -P_{1}\\ I & I \end{array}\right)\left(\begin{array}{c} e_{1}\left(\ell \right)\\ e_{1}\left(0\right) \end{array}\right)$ lies in $D_{bc}^{⊥}$. Now $D_{bc}$ is a Dirac structure and the matrix in front of $\left(\begin{array}{c} e_{1}\left(\ell \right)\\ e_{1}\left(0\right) \end{array}\right)$ equals $\sqrt{2}R_{0}$, and so we conclude that

$$R_{0}\left(\begin{array}{c} e_{1}\left(\ell \right)\\ e_{1}\left(0\right) \end{array}\right)\in D_{bc}^{⊥}=D_{bc}=\mathrm{ran}\left(\begin{array}{c} F\\ E \end{array}\right).$$

So, we have shown that ${\left(D\circ D_{bc}\right)}^{⊥}\subseteq D\circ D_{bc}$. Combining this with the result obtained in the first part of the proof, we conclude that $D\circ D_{bc}$ is a Dirac structure. □

As is clear from Figure 9, $D\circ D_{bc}$ is the interconnection of the Dirac structure D and the Dirac structure $D_{bc}$. If both Dirac structures were finite-dimensional, then there is a general result stating that the interconnection is again a Dirac structure, see e.g., [17,18]. Unfortunately, such a result does not hold in general, see [9], but it holds if one of the two is finite-dimensional [19]. The latter is our case, but we have chosen to present a direct proof.

Combining Theorem 2 with Theorem 1 gives the following corollary.

**Corollary** **1.** *Under the conditions of Theorem 2, the operator $J:\mathrm{dom}\left(J\right)\subset L^{2}\left(\left(0,\ell \right);R^{n}\right)\mapsto L^{2}\left(\left(0,\ell \right);R^{n}\right)$ given by*

(43) $$Je=P_{1}\frac{de}{d\zeta }+P_{0}e$$

*with*

(44) $$\mathrm{dom}\left(J\right)=\left\{e\in H^{1}\left(\left(0,\ell \right);R^{n}\right)|R_{0}\left(\begin{array}{c} e(\ell )\\ e(0) \end{array}\right)\in \mathrm{ran}\left(\begin{array}{c} F\\ E \end{array}\right)\right\}$$

*is skew-adjoint.*

Based on Lemma 2 and Exercise 3, the condition on $\left(\begin{array}{c} e(\ell )\\ e(0) \end{array}\right)$ in the domain of *J* can equivalently be written as

(45) $$\left(\begin{array}{cc} E^{T}\; & F^{T} \end{array}\right)R_{0}\left(\begin{array}{c} e(\ell )\\ e(0) \end{array}\right)=0,\;or\;as\left(\begin{array}{cc} I+\Theta & I-\Theta \end{array}\right)R_{0}\left(\begin{array}{c} e(\ell )\\ e(0) \end{array}\right)=0$$

with Θ unitary (depending on *E* and *F*).

As seen in Section 3.3, Corollary 1 implies that the PDE built from composing *J* from (43) with (44) (or equivalently (45)) with a strictly positive Hamiltonian will have a unique weak solution for any initial condition. This composition is depicted in Figure 10. From this figure, we see that we have closed the Dirac structure D by imposing boundary conditions via $D_{bc}$ and linked it via the Hamiltonian density H to a state trajectory. In this way, we have built a closed system. Note that we can also build an open system, see Section 3.7, but also then we have to specify a part of the boundary conditions, i.e, choose the inputs, see also the discussion above Theorem 2.

Since a PDE normally does not have its boundary conditions given as being elements of a range, but as lying in the kernel of some matrix, the formulation with (45) is chosen. However, this formulation asks to rewrite the boundary conditions using $R_{0}$, and some E,F, or Θ having certain properties. This is possible, but it may feel obsolete. To understand the alternative formulation, we pose two questions for the PDE build from (43) and (45), i.e.,

(46) $$\frac{\partial x}{\partial t}\left(\zeta ,t\right)=P_{1}\frac{\partial }{\partial \zeta }\left[H(\zeta )x(\zeta ,t)\right]+P_{0}H\left(\zeta \right)x\left(\zeta ,t\right),\;\zeta \in \left(0,\ell \right),t\geq 0,$$

with *boundary condition*

(47) $$\left(\begin{array}{cc} E^{T}\; & F^{T} \end{array}\right)R_{0}\left(\begin{array}{c} H(\ell )x(\ell ,t)\\ H(0)x(0,t) \end{array}\right)=0,\;t\geq 0.$$

**Question** **7.** *Consider the PDE (46) with boundary condition (47).*

*1.*  *How many (independent) boundary conditions does the PDE have?*
*2.*  *Define the Hamiltonian $H\left(t\right):=\frac{1}{2}\langle x\left(t\right),Hx\left(t\right)\rangle$. What do you know about $\dot{H}\left(t\right)$?*

Based on the insight gained from the above question, the third bullet could be added to the following theorem, see [2]. The equivalence between the first two bullets can be found in [6], and follows basically from Corollary 1 and the results as described in Section 3.3.

**Theorem** **3.** *Given the port-Hamiltonian partial differential Equation (46) with $P_{1},P_{0},H\left(\zeta \right)\in R^{n\times n}$, $P_{1}$ invertible and symmetric, $P_{0}$ skew-symmetric, H(ζ) symmetric satisfying 0<mI≤H(ζ)≤MI. The boundary conditions are given as $W_{B}R_{0}\left(\begin{array}{c} H(\ell )x(\ell ,t)\\ H(0)x(0,t) \end{array}\right)=0$, with $W_{B}$ a n×2n-matrix. Then the following are equivalent:*

- *The PDE has for every $x_{0}\in X$ a unique weak solution satisfying ${∥x\left(t\right)∥}_{H}={∥x_{0}∥}_{H}$, t∈R, where ${∥\cdot ∥}_{H}^{2}=\frac{1}{2}\langle \cdot ,H\cdot \rangle$;*
- *$W_{B}$ can be written as $S\left(\begin{array}{cc} I+\Theta & \;I-\Theta \end{array}\right)$ with S invertible and* Θ *unitary;*
- *$W_{B}$ has full rank, and $\dot{H}\left(0\right)=0$ for all (smooth) initial conditions satisfying the boundary conditions.*

Since $R_{0}$ is invertible, the last item can equivalently be formulated as:

- *${\tilde{W}}_{B}:=W_{B}R_{0}$ has full rank, and $\dot{H}\left(0\right)=0$ for all (smooth) initial conditions satisfying the boundary conditions.*

In this way the condition is directly formulated in terms of the matrix in front of $\left(\begin{array}{c} H(\ell )x(\ell ,t)\\ H(0)x(0,t) \end{array}\right)$, which is more confinient as can also be seen from the following example.

### 3.5. Example

As our (running) example we consider the *vibrating string* as shown in Figure 11.

The PDE describing the (undamped) vibrating string is

$$\frac{\partial^{2}w}{\partial t^{2}}\left(\zeta ,t\right)=\frac{1}{\rho (\zeta )}\frac{\partial }{\partial \zeta }\left[T\left(\zeta \right)\frac{\partial w}{\partial \zeta }\left(\zeta ,t\right)\right],$$

where w(ζ,t) is the deviation from the rest state at position ζ and time *t*, ρ the mass density, and *T* Young’s modulus.

We choose $x_{1}:=\rho \frac{\partial w}{\partial t}$ (the momentum), $x_{2}:=\frac{\partial w}{\partial \zeta }$ (the strain), and write the PDE as, see also Example 7,

$$\frac{\partial }{\partial t}\left(\begin{array}{c} x_{1}\\ x_{2} \end{array}\right)\left(\zeta ,t\right)=\underset{J}{\underbrace{\underset{=P_{1}}{\underbrace{\left(\begin{array}{cc} 0 & 1\\ 1 & 0 \end{array}\right)}}\frac{\partial }{\partial \zeta }}}\left[\underset{=H}{\underbrace{\left(\begin{array}{cc} \frac{1}{\rho (\zeta )} & 0\\ 0 & T(\zeta ) \end{array}\right)}}x\left(\zeta ,t\right)\right].$$

As is clear from Figure 11, our vibrating string is *fixed* at ζ=0 and moves *freely* at ζ=ℓ. In our state variables it becomes

(48) $$\rho {\left(0\right)}^{-1}x_{1}\left(0,t\right)=0\;and\;T\left(\ell \right)x_{2}\left(\ell ,t\right)=0,$$

i.e., zero velocity at the left hand side and no force at the other end.

The energy/Hamiltonian for this system is given by

$$H\left(t\right)=\frac{1}{2}\int_{0}^{\ell }x{\left(\zeta ,t\right)}^{⊤}H\left(\zeta \right)x\left(\zeta ,t\right)d\zeta =\langle x\left(\cdot ,t\right),Hx\left(\cdot ,t\right)\rangle ,$$

i.e., kinetic plus potential energy.

Now we check the conditions of Theorem 3.

- $P_{1}=\left(\begin{array}{cc} 0 & 1\\ 1 & 0 \end{array}\right)$ is an invertible 2×2 matrix (n=2).
- $P_{0}=0$, so skew-symmetric.
- If $0<m\leq T\left(\zeta \right),\rho {\left(\zeta \right)}^{-1}\leq M$ for all ζ, then $H\left(\zeta \right)=\left(\begin{array}{cc} \rho {\left(\zeta \right)}^{-1} & 0\\ 0 & T(\zeta ) \end{array}\right)$ satisfies $mI_{2}\leq H\left(\zeta \right)\leq MI_{2}$.

So we see that the general conditions in Theorem 3 are satisfied to conclude existence of solution, we can either check the second or the third bullet in that theorem. We choose the third one.

- The boundary conditions (48) are of the form $W_{B}R_{0}\left(\begin{array}{c} H(\ell )x(\ell ,t)\\ H(0)x(0,t) \end{array}\right)=0$ for $W_{B}=\left(\begin{array}{cccc} 0 & 1 & 0 & 0\\ 0 & 0 & 1 & 0 \end{array}\right)R_{0}^{-1}=\frac{1}{\sqrt{2}}\left(\begin{array}{cccc} 1 & 0 & 0 & 1\\ 0 & -1 & 1 & 0 \end{array}\right)$. This matrix has clearly rank 2.
- We have, see also Lemma 4, that along smooth solutions,
  $$\begin{array}{cc} \dot{H}\left(t\right)= & \frac{1}{2}{\left[{\left(Hx\right)}^{T}\left(\zeta ,t\right)P_{1}\left(Hx\right)\left(\zeta ,t\right)\right]}_{0}^{\ell }\\ = & \frac{1}{2}{\left[{\left(\begin{array}{c} \frac{1}{\rho (\zeta )}x_{1}\left(\zeta ,t\right)\\ T\left(\zeta \right)x_{2}\left(\zeta ,t\right) \end{array}\right)}^{T}\left(\begin{array}{cc} 0 & 1\\ 1 & 0 \end{array}\right)\left(\begin{array}{c} \frac{1}{\rho (\zeta )}x_{1}\left(\zeta ,t\right)\\ T\left(\zeta \right)x_{2}\left(\zeta ,t\right) \end{array}\right)\right]}_{0}^{\ell }. \end{array}$$
  Applying the boundary conditions (48) this equals zero.

Thus our vibrating string has for every $x_{0}\in X$ a unique weak solution for t∈R with constant energy.

**Exercise** **14.** *Rewrite $W_{B}$ in the above example as $S\left(\begin{array}{cc} I+\Theta & \;I-\Theta \end{array}\right)$ with S invertible and* Θ *unitary. Conclude from that the existence of unique weak solutions with constant energy.*

In this example we showed that every solution maintained its initial energy. However, it very likely that this would not be the case if there would be a damping at the right hand side of the vibrating in Figure 11. In that case we would still expect existence of solutions, but now with decaying energy. This will be shown in the next subsection.

### 3.6. Contractive Solution to pH-PDE

Consider that in Figure 10 we added a damping to the left hand side of $D_{bc}$, see Figure 12. In that case the Dirac structure $D_{bc}$ in Figure 10 must be replaced by a Dirac structure (with the same name) in the variables $\left(f_{\partial },f_{1},e_{\partial },e_{1}\right)$ and the connection to the *R*-component is done via $f_{1}=\phi$ and $e_{1}=R\phi$ with *R* a square matrix.

**Question** **8.** *If $R\in R^{m\times m}$ is symmetric and non-negative, what would now hold for $\dot{H}\left(t\right)$?*

Based on the insight from Question 8 and the formulation of Theorem 3 the equivalence between the first and the third bullet in the following theorem is not so surprising.

**Theorem** **4** **([2,6]).** *Given our port-Hamiltonian partial differential equation, (46),*

$$\frac{\partial x}{\partial t}\left(\zeta ,t\right)=\left(P_{1}\frac{\partial }{\partial \zeta }+P_{0}\right)\left[H(\zeta )x(\zeta ,t)\right]$$

*with $P_{1},P_{0},H\left(\zeta \right)\in R^{n\times n}$, $P_{1}$ invertible and symmetric, $P_{0}$ skew-symmetric, H(ζ) symmetric satisfying 0<mI≤H(ζ)≤MI. The boundary conditions are given as $W_{B}R_{0}\left(\begin{array}{c} H(\ell )x(\ell ,t)\\ H(0)x(0,t) \end{array}\right)=0$, with $W_{B}$ a n×2n-matrix. Then the following are equivalent:*

- *The PDE has for every $x_{0}\in X$ a unique weak solution satisfying ${∥x\left(t\right)∥}_{H}\leq {∥x_{0}∥}_{H}$, t≥0, i.e, a *contraction *semigroup;*
- *$W_{B}$ can be written as $S\left(\begin{array}{cc} I+V & \;I-V \end{array}\right)$ with S invertible and V satisfies $VV^{⊤}\leq I$;*
- *$W_{B}$ has *full rank*, and $\dot{H}\left(0\right)\leq 0$ for all (smooth) initial conditions satisfying the boundary conditions.*

**Exercise** **15.** *Consider the vibrating string of Figure 13. Model the damper as a linear relation between the velocity and the force at the end- tip, and show that for every initial condition with finite energy there exists a unique weak solution.*

Until now we have only considered homogeneous PDE, i.e., there are no inputs nor outputs. In the next subsection we present general results on existence of solutions for our class of PDEs with inhomogeneous boundary conditions.

### 3.7. Input and Outputs, Inhomogeneous pH-PDEs

We take our port- Hamiltonian PDE from the previous subsection, but add an input, i.e., make it inhomogeneous. Furthermore we add an (boundary) measurement. Using the graphical representation of Figure 12, this is graphically shown in Figure 14.

The corresponding partial differential equation is

(49) $$\begin{array}{cc} \frac{\partial x}{\partial t}\left(\zeta ,t\right)= & \;\left(P_{1}\frac{\partial }{\partial \zeta }+P_{0}\right)\left[H(\zeta )x(\zeta ,t)\right]; \end{array}$$

(50) $$\begin{array}{cc} 0= & \;W_{B,1}\left(\begin{array}{c} f_{\partial }\left(t\right)\\ e_{\partial }\left(t\right) \end{array}\right); \end{array}$$

(51) $$\begin{array}{cc} u(t)= & \;W_{B,2}\left(\begin{array}{c} f_{\partial }\left(t\right)\\ e_{\partial }\left(t\right) \end{array}\right); \end{array}$$

(52) $$\begin{array}{cc} y(t)= & \;W_{C}\left(\begin{array}{c} f_{\partial }\left(t\right)\\ e_{\partial }\left(t\right) \end{array}\right). \end{array}$$

with $\left(\begin{array}{c} f_{\partial }\left(t\right)\\ e_{\partial }\left(t\right) \end{array}\right)=R_{0}\left(\begin{array}{c} H(\ell )x(\ell ,t)\\ H(0)x(0,t) \end{array}\right)$, $W_{B,1}\in R^{n-m,2n}$, $W_{B,2}\in R^{m,2n}$, and $W_{C}\in R^{k,2n}$.

**Theorem** **5** **([2,20]).** *Given the port-Hamiltonian partial differential Equation (49) with boundary conditions, input and outputs (50)–(52). Assume the (standard) assumptions on $P_{1}$, $P_{0}$ and H and assume further that $W_{B}:=\left(\begin{array}{c} W_{B,1}\\ W_{B,2} \end{array}\right)$ is a *full rank *matrix.*

*If there exists a unique weak solution when u≡0, then for every initial condition in X and every $u\in L^{2}\left(\left(0,t_{1}\right);R^{m}\right)$ there is a unique solution with $y\in L^{2}\left(\left(0,t_{1}\right);R^{k}\right)$, $t_{1}>0$ arbitrary.*

In Theorems 3 and 4 we have given simple conditions for existence of solutions to the homogeneous PDE, i.e., when u≡0, and so the conditions in the above theorem are not hard to check. The novelty of this theorem lies in the large class of input functions which we may choose. For smooth input functions, existence of the inhomogeneous PDE is not hard to show, see for this class of PDEs e.g., [6]. The proof of Theorem 5 is quite involved and relies heavily on a result by G. Weiss [21].

**Example** **8.** *Consider the vibrating string with control and observation at the right tip, as shown in Figure 15.*

*We assume that we control the force and measure the velocity at the right hand side. Hence our PDE becomes*

$$\begin{array}{cc} \frac{\partial^{2}w}{\partial t^{2}}\left(\zeta ,t\right) & =\frac{1}{\rho (\zeta )}\frac{\partial }{\partial \zeta }\left[T\left(\zeta \right)\frac{\partial w}{\partial \zeta }\left(\zeta ,t\right)\right]\\ \frac{\partial w}{\partial t}\left(0,t\right) & =0,\;T\left(\ell \right)\frac{\partial w}{\partial \zeta }\left(\ell ,t\right)=u\left(t\right)\\ \frac{\partial w}{\partial t}\left(\ell ,t\right) & =y(t). \end{array}$$

*If we take the controlling force equal to zero, then the above PDE has the homogenous boundary conditions as the one in Section 3.5. There we showed that this has a unique (weak) solution (even a unitary group). Hence by Theorem 5, we conclude that the above inhomogeneous PDE has a unique (weak) solution for all initial conditions in X and every $u\in L^{2}\left(0,t_{1}\right)$. Furthermore, the output will be in $L^{2}\left(0,t_{1}\right)$ for all $t_{1}>0$.*

**Exercise** **16.** *In this exercise we want to link the previous example back to its Dirac structure.*

*1.*  *Identify for the partial differential equation of Example 8 the Dirac structure as in Figure 8. That is, find $P_{1},P_{0}$ and $f_{\partial },e_{\partial }$.*
*2.*  *Use the zero power balance of the Dirac structure, see (6), to conclude that for the partial differential equation of Example 8 there holds*
$$\dot{H}\left(t\right)=u\left(t\right)y\left(t\right).$$
*3.*  *Check the above equality directly on the model of Example 8.*

### 3.8. Transfer Functions

Now that we have introduced for our linear PDEs inputs and outputs, the concept of a transfer function is a logical continuation. We start by its general definition. Since a transfer function will be complex valued, it is necessary to assume that our spaces are complex linear spaces.

**Definition** **4.** *Let* Σ *be a system with input u(t), output y(t) and remaining variables z(t).*

*Let s∈C and $u_{0}\in U$ (input (value) space) be given. If there exists a solution ${\left(u\left(t\right),z\left(t\right),y\left(t\right)\right)}_{t\geq 0}$ of the form $\left(u\left(t\right),z\left(t\right),y\left(t\right)\right)={\left(u_{0}e^{st},z_{0}e^{st},y_{0}e^{st}\right)}_{t\geq 0}$, then this is called an *exponential solution.

*If, with the s∈C fixed, for every $u_{0}\in U$, there exists a (unique) exponential solution, then the map G(s):U↦Y, $G\left(s\right)u_{0}=y_{0}$ is called the *transfer function at *s of the system* Σ.

We apply this definition to the pH-PDE of Equations (49)–(52). So we take an $u_{0}\in C^{m}$, s∈C and try to find an exponential solution to these equations. Substituting the candidate exponential solution into the equations gives

$$\begin{array}{cc} \frac{\partial }{\partial t}\left[x_{0}\left(\zeta \right)e^{st}\right]= & \;\left(P_{1}\frac{\partial }{\partial \zeta }+P_{0}\right)\left[H\left(\zeta \right)x_{0}\left(\zeta \right)e^{st}\right];\\ 0= & \;W_{B,1}\left(\begin{array}{c} f_{\partial ,0}e^{st}\\ e_{\partial ,0}e^{st} \end{array}\right);\;u_{0}e^{st}=W_{B,2}\left(\begin{array}{c} f_{\partial ,0}e^{st}\\ e_{\partial ,0}e^{st} \end{array}\right);\\ y_{0}e^{st}= & \;W_{C}\left(\begin{array}{c} f_{\partial ,0}e^{st}\\ e_{\partial ,0}e^{st} \end{array}\right). \end{array}$$

Trivially, we have that $\frac{\partial }{\partial t}\left[x_{0}\left(\zeta \right)e^{st}\right]=sx_{0}\left(\zeta \right)e^{st}$ and so in the above equations every term contains an $e^{st}$. Since $e^{st}\neq 0$ for all s∈C and all t∈R, we see that solving the above is the same as solving

$$\begin{array}{cc} sx_{0}\left(\zeta \right)= & \;\left(P_{1}\frac{d}{d\zeta }+P_{0}\right)\left[H\left(\zeta \right)x_{0}\left(\zeta \right)\right];\\ 0= & \;W_{B,1}\left(\begin{array}{c} f_{\partial ,0}\\ e_{\partial ,0} \end{array}\right);\;u_{0}=W_{B,2}\left(\begin{array}{c} f_{\partial ,0}\\ e_{\partial ,0} \end{array}\right);\\ y_{0}= & \;W_{C}\left(\begin{array}{c} f_{\partial ,0}\\ e_{\partial ,0} \end{array}\right). \end{array}$$

Although this is a linear ODE it is most-times very hard to solve. This is mainly due to the non-constant coefficients in the differential equation. However, the balance equation can give properties of the transfer function G(s), as we shall show in more detail with an example.

**Example** **9.** *We consider the system of Example 8. That is*

$$\begin{array}{cc} \frac{\partial^{2}w}{\partial t^{2}}\left(\zeta ,t\right) & =\frac{1}{\rho (\zeta )}\frac{\partial }{\partial \zeta }\left[T\left(\zeta \right)\frac{\partial w}{\partial \zeta }\left(\zeta ,t\right)\right],\;\zeta \in \left(0,\ell \right),t\geq 0,\\ \frac{\partial w}{\partial t}\left(0,t\right) & =0,\;T\left(\ell \right)\frac{\partial w}{\partial \zeta }\left(\ell ,t\right)=u\left(t\right),\\ \frac{\partial w}{\partial t}\left(\ell ,t\right) & =y(t). \end{array}$$

*Since for the transfer function we are working with complex valued signals, we extend the energy to these signals. We have*

(53) $$H\left(t\right)=\frac{1}{2}\int_{0}^{\ell }\rho \left(\zeta \right){\left|\frac{\partial w}{\partial t}\left(\zeta ,t\right)\right|}^{2}+T\left(\zeta \right){\left|\frac{\partial w}{\partial \zeta }\left(\zeta ,t\right)\right|}^{2}d\zeta .$$

*It is not hard to see that this equals 〈x(·,t),H(·)x(·,t)〉 where 〈f,g〉 is the (complex) inner product on $L^{2}\left(\left(0,\ell \right);C^{n}\right)$. We have, see before,*

H˙(t)=12T(ℓ)∂w∂ζ(ℓ,t)¯∂w∂t(ℓ,t)−T(0)∂w∂ζ(0,t)¯∂w∂t(0,t)+T(ℓ)∂w∂ζ(ℓ,t)∂w∂t(ℓ,t)¯−T(0)∂w∂ζ(0,t)∂w∂t(0,t)¯=12u(t)∗y(t)+u(t)y(t)∗=Re(u(t)∗y(t)).

*So for the exponential solution*

H˙(t)=ddt〈x0est,Hx0est〉=Re(u0∗es∗ty0est),

*or equivalently,*

s〈x0est,Hx0est〉+s∗〈x0est,Hx0est〉=Re(u0∗es∗ty0est).

*Now es∗test=e2Re(s)t, and we can divide both sides of the equality by this real exponential. This gives*

2Re(s)〈x0,Hx0〉=Re(u0∗y0)=Re(u0∗G(s)u0)=Re(G(s))|u0|2.

*Since $\langle x_{0},Hx_{0}\rangle \geq 0$, we find Re(G(s))≥0 for Re(s)>0. This is known as G being positive real.*

So the previous example shows that even without calculating the transfer function, we can say something about it. As you have seen this is a direct consequence of the balance equation. Since the balance equation is coming from the underlying Dirac structure, we see that the influence of this Dirac structure is huge.

## Figures and Tables

**Figure 5:**
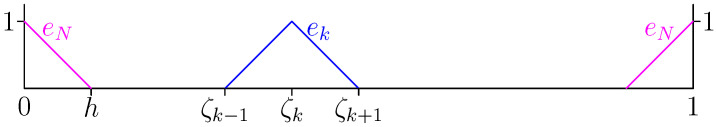
The hat-functions, $e_{k}$.

**Figure 7:**
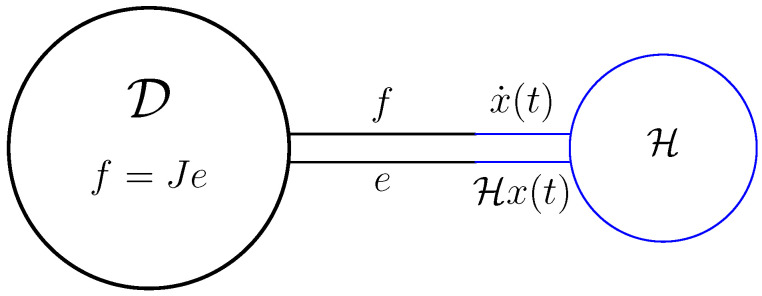
The Dirac structure connected to the storage H.

**Figure 8:**
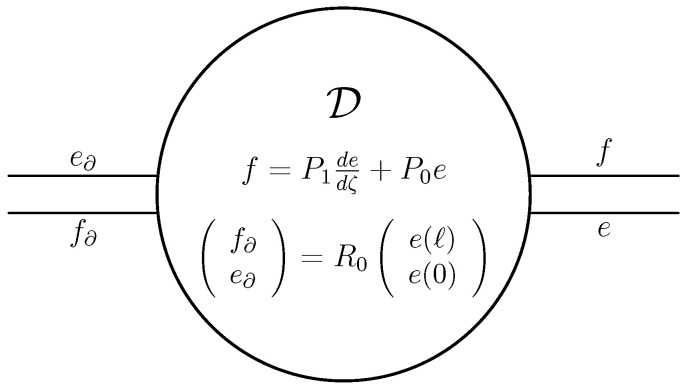
The Dirac structure associated to (39).

**Figure 9:**
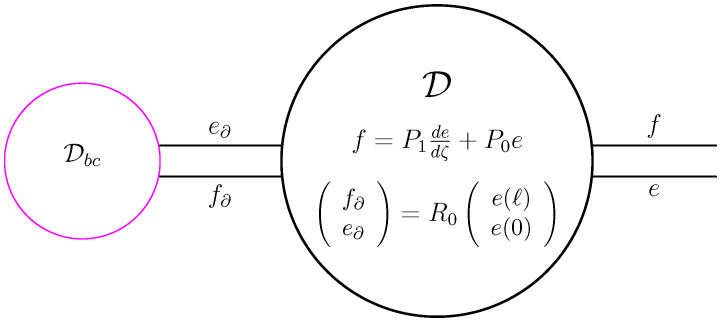
The Dirac structure associated to (40).

**Figure 10:**
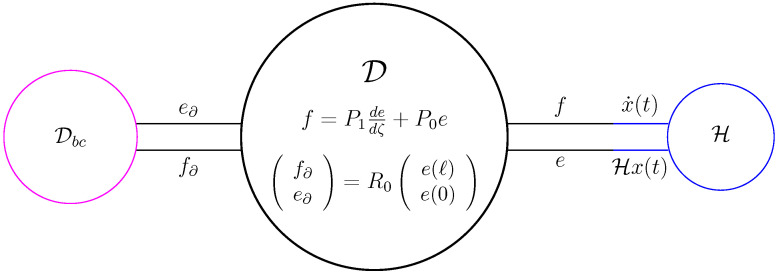
The interconnection of the Dirac structure (40) with the Hamiltonian H.

**Figure 11:**
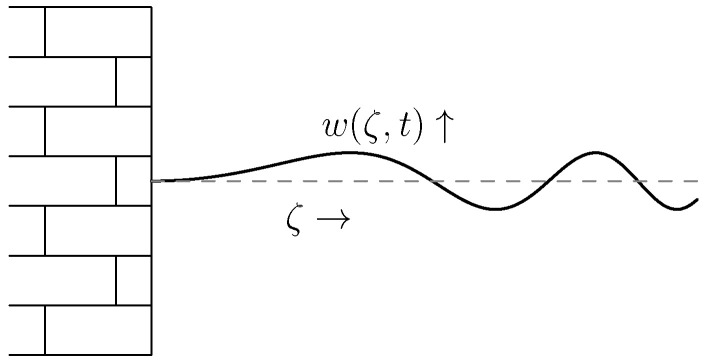
String vibrating around it rest position (dashed line).

**Figure 12:**
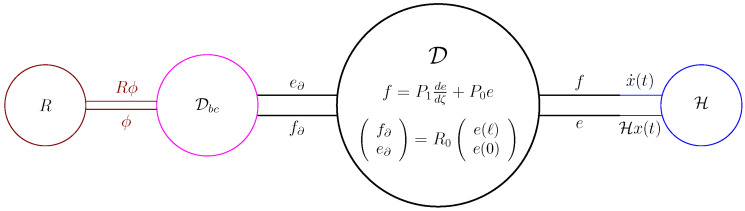
A pH system with damping.

**Figure 13:**
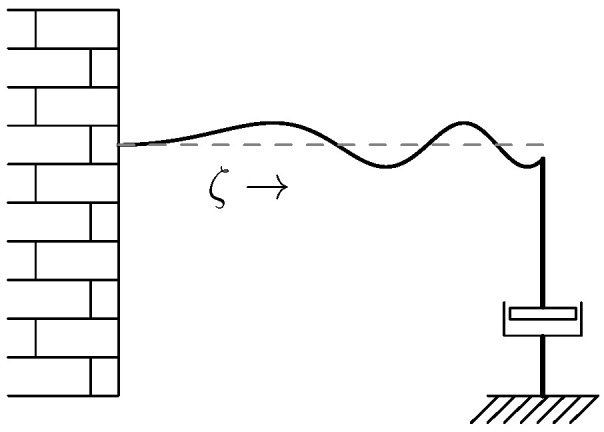
Vibrating string with damper.

**Figure 14:**
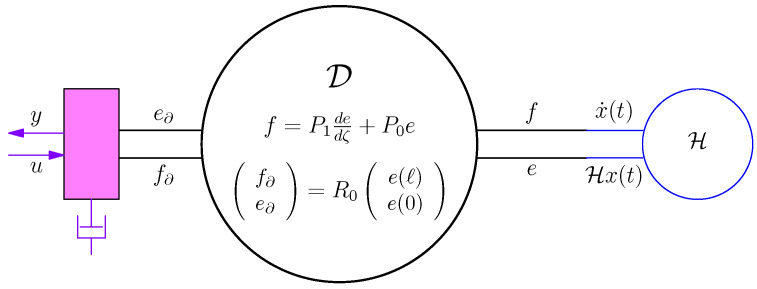
Our pH system with damping, inputs, and outputs.

**Figure 15:**
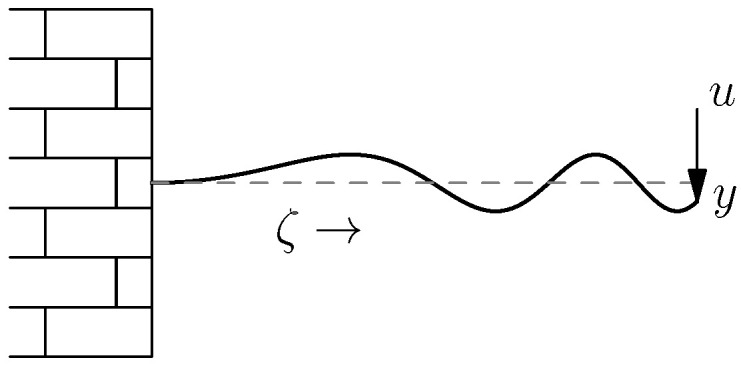
Vibrating string with control and observation.

## Data Availability

No new data were created.

## Appendix A. Bond Spaces and Dual Spaces

In many papers on Dirac structures we can find that E and F are taken to be the dual of each other. However, as we have previously seen, for instance in Section 2.5, Section 2.6 and Section 2.7, this is not needed. Nevertheless, there is a relation between F and the dual of E. Namely, as will shall show in the appendix, F can always be regarded as a subspace of the dual of E.

Let the bond space B=F×E with the bilinear relation 〈f∣e〉 be given, define (for fixed f∈F) the map

$$\ell_{f}:E\mapsto R\;as\;\ell_{f}\left(e\right)=\langle f|e\rangle .$$

Then, this is a linear map from E to R. Thus, there exists a (unique) element $\varepsilon \in E^{\prime }$ (the *algebraic dual* of E) such that

$$\langle f|e\rangle ={\langle \varepsilon ,e\rangle }_{E^{\prime }\times E}.$$

Hence, we can define the “identification” map

(A1) $$Id:F\mapsto E^{\prime }\;as\;Id\left(f\right)=\varepsilon .$$

So F can be interpreted/identified as a subspace of $E^{\prime }$. Similarly, we can interpret E as a subspace of $F^{\prime }$.

**Exercise** **A1.**
*For the map Id of Equation (A1) prove the following properties*

1. *Id is a linear map from F to $E^{\prime }$.*
2. *Id is injective if the bilinear product is non-degenerate.*

When F and E are normed, linear spaces, and the bilinear product is bounded. That is there exists an m>0 such that for all f∈F and e∈E we have

|〈f∣e〉|≤m∥f∥∥e∥.

Then the map

$$\ell_{f}:E\mapsto R\;defined\;as\;\ell_{f}\left(e\right):=\langle f|e\rangle ,$$

is a *continuous*/*bounded* linear map from E to R.

Thus there exists a (unique) element $\varepsilon \in E^{*}$ (the *topological dual* of E) such that

$$\langle f|e\rangle ={\langle \varepsilon ,e\rangle }_{E^{*}\times E}.$$

So F can be interpreted/identified as a subspace of $E^{*}$. Similarly, we can interpret E as a subspace of $F^{*}$.

## Appendix B. Abstract Differential Equation and *C*_0_-Semigroups

### Appendix B.1. Strongly Continuous Semigroups

Consider a linear, time invariant differential equation on the Hilbert (or Banach) space *X*. Assume that for every $x_{0}\in X$ there exists a (weak) solution denoted by x(t). Furthermore, assume that this solution depends continuously on the initial condition.

Define for t≥0 the map T(t):X↦X as

(A2) $$T\left(t\right)x_{0}=x\left(t\right).$$

Then it has the following properties:

- T(0)=I;
- $T\left(t_{1}+t_{2}\right)=T\left(t_{1}\right)T\left(t_{2}\right)$, $t_{1},t_{2},\in \left[0,\infty \right)$, *time-invariance*;
- T(t) is for every t≥0 a linear and bounded operator, i.e., T(t)∈L(X).

These properties lead to the following definition

**Definition** **A1.** *${\left(T(t)\right)}_{t\geq 0}$ is a strongly continuous semigroup, or short* $C_{0}$-semigroup *if it satisfies the above three properties and for all $x_{0}\in X$*

(A3) $$\lim_{t↓0}∥T\left(t\right)x_{0}-x_{0}∥=0.$$

So, in terms of the solution x(t), see (A2), the condition (A3) demands that the solution is continuous at t=0. It is not hard to show that on $X=R^{n}$ the exponential $e^{At}$ is a $C_{0}$-semigroup.

**Exercise** **A2.**
*Show that the solution map of the PDE*

$$\frac{\partial w}{\partial t}\left(\zeta ,t\right)=\frac{\partial w}{\partial \zeta }\left(\zeta ,t\right),\;w\left(\ell ,t\right)=0,\;w\left(\zeta ,0\right)=w_{0}\left(\zeta \right).$$

*is a $C_{0}$-semigroup on $x=L^{2}\left(0,\ell \right)$.*

*Hint: See Exercise 13.*

Since T(t) came from x(t) via $x\left(t\right)=T\left(t\right)x_{0}$, we have

$$\dot{x}\left(t\right)=\lim_{h↓0}\frac{x(t+h)-x(t)}{h}=\lim_{h↓0}\frac{T\left(t+h\right)x_{0}-T\left(t\right)x_{0}}{h}.$$

Thus, by the semigroup and boundedness property,

(A4) $$\dot{x}\left(t\right)=\lim_{h↓0}\frac{T\left(t\right)T\left(h\right)x_{0}-T\left(t\right)x_{0}}{h}=T\left(t\right)\lim_{h↓0}\frac{T\left(h\right)x_{0}-x_{0}}{h}.$$

We define (*whenever it exists*)

(A5) $$Ax_{0}:=\lim_{h↓0}\frac{T\left(h\right)x_{0}-x_{0}}{h}.$$

With this and (A4) we obtain that x(t) satisfies the (abstract) differential equation

(A6) $$\dot{x}\left(t\right)=T\left(t\right)Ax_{0}=AT\left(t\right)x_{0}=Ax\left(t\right).$$

### Appendix B.2. Abstract Differential Equation

In the previous subsection we have seen that a $C_{0}$-semigroup leads to an abstract differential equation. Of course the reverse question is much more interesting; Given the linear operator A:dom(A)⊆X↦X, under which conditions does the abstract differential equation

(A7) $$\dot{x}\left(t\right)=Ax\left(t\right),\;x\left(0\right)=x_{0}$$

have solution, i.e., when do we have the existence of a $C_{0}$-semigroup?

For *X* being a Hilbert space (*from now on standard assumption*) we have the following important theorems.

**Theorem** **A1.**
*If A is skew-adjoint, i.e., $A^{*}=-A$, then A generates a $C_{0}$-semigroup satisfying*

- *∥T(t)∥=1 for all t≥0;*
- *T(t) can be extended to the whole real time, and $T\left(t_{1}+t_{2}\right)=T\left(t_{1}\right)T\left(t_{2}\right)$, $t_{1},t_{2}\in R$ and ∥T(t)∥=1 for all t∈R.*

A $C_{0}$-semigroup satisfying the two “bullets” in the above theorem is named a *unitary group*.

**Theorem** **A2.** *If A is densely defined, closed, and *dissipative*, i.e., 〈Ax,x〉≤0 ∀x∈dom(A), and if $A^{*}$ is dissipative, then A generates a $C_{0}$-semigroup satisfying ∥T(t)∥≤1 for all t≥0, i.e., a contraction semigroup.*

From (A6) we are temped to conclude that $x\left(t\right)=T\left(t\right)x_{0}$ is the solution of (A7), but care should be taken. If $x_{0}\in \mathrm{dom}\left(A\right)$ the function $x\left(t\right)=T\left(t\right)x_{0}$ a *classical* solution, i.e., $T\left(t\right)x_{0}\in \mathrm{dom}\left(A\right)$ for all t≥0, x(t) is differentiable, and (A7) holds pointwise. For $x_{0}\in X$ it is a *weak* or *mild* solution, i.e., for every $z\in \mathrm{dom}(A^{*})$ and $t_{f}>0$ there holds (compare to (36)),

$$\langle z,x\left(t_{f}\right)\rangle -\langle z,x_{0}\rangle =\int_{0}^{t_{f}}\langle A^{*}z,x\left(t\right)\rangle dt.$$

The above two theorems are very frequently used to prove existence of solutions. The following lemma is also useful in this.

**Lemma** **A1.**
*Let X be a Hilbert space with inner product 〈·,·〉 and let Q∈L(X) satisfying $Q=Q^{*}$ and $\langle x,Qx\rangle \geq {m∥x∥}^{2}$, ∀x∈X.*

1. *If J is skew-adjoint in X, then JQ is skew-adjoint in the inner product ${\langle x,z\rangle }_{Q}:=\langle x,Qz\rangle$.*
2. *If A is densely defined and dissipative in X, then AQ is densely defined and it is dissipative in the inner product ${\langle x,z\rangle }_{Q}:=\langle x,Qz\rangle$.*

**Exercise** **A3.**
*Prove the above lemma.*
